# MicroRNA-593-5p contributes to cell death following exposure to 1-methyl-4-phenylpyridinium by targeting PTEN-induced putative kinase 1

**DOI:** 10.1016/j.jbc.2023.104709

**Published:** 2023-04-14

**Authors:** Myungsik Yoo, Doo Chul Choi, Aleta Murphy, Atiq M. Ahsan, Eunsung Junn

**Affiliations:** Department of Neurology, RWJMS Institute for Neurological Therapeutics, Rutgers -Robert Wood Johnson Medical School, Piscataway, New Jersey, USA

**Keywords:** microRNA, neurodegeneration, PTEN-induced putative kinase 1, cell death, molecular cell biology

## Abstract

Neurodegenerative diseases are characterized by a decline in neuronal function and structure, leading to neuronal death. Understanding the molecular mechanisms of neuronal death is crucial for developing therapeutics. MiRs are small noncoding RNAs that regulate gene expression by degrading target mRNAs or inhibiting translation. MiR dysregulation has been linked to many neurodegenerative diseases, but the underlying mechanisms are not well understood. As mitochondrial dysfunction is one of the common molecular mechanisms leading to neuronal death in many neurodegenerative diseases, here we studied miRs that modulate neuronal death caused by 1-methyl-4-phenylpyridinium (MPP^+^), an inhibitor of complex I in mitochondria. We identified miR-593-5p, levels of which were increased in SH-SY5Y human neuronal cells, after exposure to MPP^+^. We found that intracellular Ca^2+^, but not of reactive oxygen species, mediated this miR-593-5p increase. Furthermore, we found the increase in miR-593-5p was due to enhanced stability, not increased transcription or miR processing. Importantly, we show the increase in miR-593-5p contributed to MPP^+^-induced cell death. Our data revealed that miR-593-5p inhibits a signaling pathway involving PTEN-induced putative kinase 1 (PINK1) and Parkin, two proteins responsible for the removal of damaged mitochondria from cells, by targeting the coding sequence of PINK1 mRNA. Our findings suggest that miR-593-5p contributes to neuronal death resulting from MPP^+^ toxicity, in part, by impeding the PINK1/Parkin-mediated pathway that facilitates the clearance of damaged mitochondria. Taken together, our observations highlight the potential significance of inhibiting miR-593-5p as a therapeutic approach for neurodegenerative diseases.

MiRs are short, single-stranded endogenous noncoding RNA molecules about 21 to 25 nucleotides in length (1, 2). The primary miR (pri-miR) transcript is transcribed by RNA polymerase II and goes through two cleavage events until mature miR is generated. In the first event, the RNase III Drosha associates with microprocessor complex subunit DGCR8 to cleave pri-miR into precursor miR (pre-miR). In the second, the pre-miR is transported to the cytoplasm and is cleaved by Dicer to form a mature miR, which is assembled into an RNA-induced silencing complex. By complexing with RNA-induced silencing complex, the miR 5′ seed region (between nucleotides 2 and 7) interacts with the 3′-UTR of target mRNA to induce its degradation or translation inhibition. Thus, miRs have been recognized as important posttranscriptional regulators of gene expression that play important roles in various cellular pathways. In addition, dysregulation of the generation of miR is strongly associated with the pathogenesis of diseases including neurodegenerative disorders (3, 4). The identification of miR(s) contributing to the pathogenesis of disease can provide new therapeutic avenues by either rescuing the expression of missing miR(s) or inhibiting the expression of overexpressed miR(s) (5).

Neurodegenerative disorders are characterized by the selective death of neuronal subtypes. Thus, a great deal of research has been done to understand the process of neuronal death. Mitochondrial dysfunction is a common molecular event leading to neuronal death in many neurodegenerative disorders, including Parkinson’s disease (PD) (6). For example, reduced activity of complex I (NADH dehydrogenase) in the mitochondrial electron transport chain was observed in the substantia nigra of PD patients (7), followed by studies detecting a comparable complex I deficit in the platelets of PD patients (8, 9). Consistent with this, administration of complex I inhibitors, such as 1-methyl-4-phenyl-1,2,3,6-tetrahydropyridine (MPTP) and rotenone, in animals produced neuropathological and behavioral symptoms like human PD (10, 11, 12). To better understand the pathways of neuronal cell death following mitochondrial dysfunction, we studied miRs that play important roles in this pathway. In the current study, we found that the expression of miR-593-5p increased dramatically after exposure to 1-methyl-4-phenylpyridinium (MPP^+^), an active metabolite form of MPTP, in neuronal cells. Furthermore, we showed that increased expression of miR-593-5p leads to neuronal death in part by inhibiting the pathway mediated by PINK1 and Parkin, which are involved in the removal of damaged mitochondria from cells, *via* the downregulation of PINK1 expression.

## Results

### Accumulation of miR-593-5p in human neuronal cells following MPP^+^ exposure

**Figure 1:**
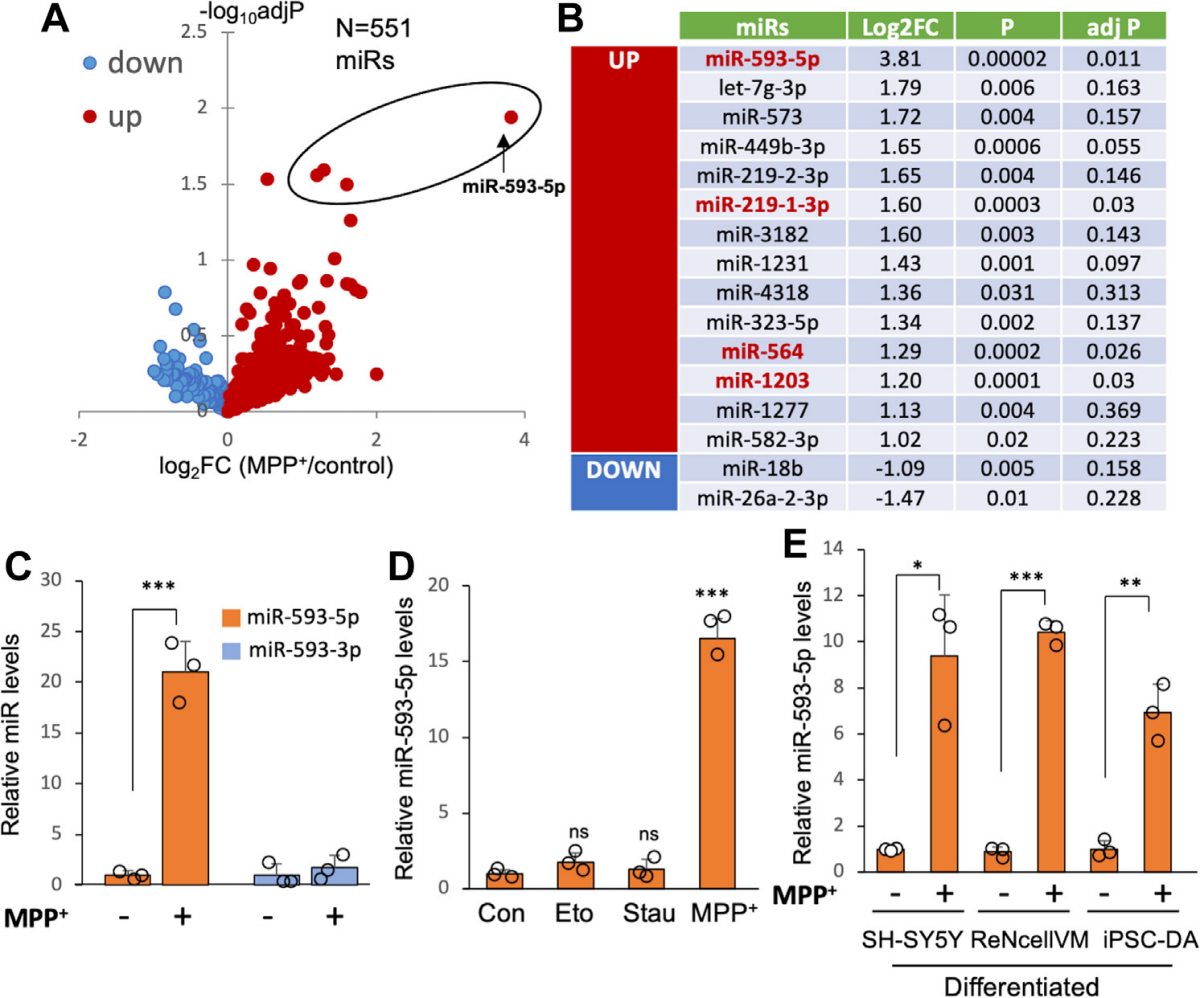
**Increased miR-593-5p expression following MPP**^**+**^**treatment.***A*, the volcano plot summarizes the results of miR profiling studies in SH-SY5Y cells. It shows upregulated miRs in *red* and downregulated miRs in *blue*. The four *red dots* within an *oval* represent miRs that were significantly increased (log2FC > 1) after MPP^+^ exposure, as determined by the Benjamini–Hochberg method (adj *p* < 0.05). *B*, list of miRs differentially expressed following MPP^+^ (log_2_FC > 1, *p* < 0.05). Of these, four miRs in an *oval* in volcano plot are shown in *red*. *C*, quantification of miR-593-5p and miR-593-3p in SH-SY5Y cells following MPP^+^ (2 mM) for 12 h. *D*, quantification of miR-593-5p in SH-SY5Y cells following etoposide (Eto, 50 μM), staurosporine (Stau, 100 nM), and MPP^+^ (2 mM) exposure for 12 h. *E*, the increase of miR-593-5p in differentiated SH-SY5Y, ReNcell VM cells, and iPSC-derived dopaminergic neurons after treatment with 0.5 mM MPP^+^ for 12 h was measured using qRT-PCR. In qRT-PCR of miRs, U6 RNA levels were used for normalization. Data are mean ± SD for three biological replicates. Each *circle* represents mean of three technical replicates. Student *t* test was performed between MPP^+^-treated samples and control samples, ∗*p* < 0.05, ∗∗*p* < 0.01, ∗∗∗*p* < 0.001. MPP^+^, 1-methyl-4-phenylpyridinium.

Mitochondrial dysfunction has been implicated as a cause of neurodegenerative diseases, including PD (6). Therefore, we aimed to identify specific miRs that contribute to neuronal death caused by mitochondrial dysfunction. To do this, we treated SH-SY5Y cells with 2 mM MPP^+^ for 12 h and performed miR profiling studies using “Human miRNome miScript miRNA PCR Array” (Qiagen). Of the 1066 miRs in the array, low expressing miRs (>Ct 30) in both control- and MPP^+^-treated samples were removed for analysis. Out of the remaining 551 miRs, four miRs were found to be differentially expressed using the criterion of >2-fold change, adj *p* < 0.05 (Fig. 1, *A* and *B* and Table S1). Among these, miR-593-5p exhibited a dramatic increase in MPP^+^-treated cells. In contrast, levels of miR-593-3p, which is produced from the same precursor miR-593 (pre-miR-593), did not change (Fig. 1*C*). The significant increase in miR-593-5p observed 12 h after MPP^+^ exposure does not appear to be a direct result of cell death, as there was only a low amount of cell death (≤10%) at this time point (Fig. S1). Furthermore, miR-593-5p expression did not change in response to other cell death inducers, such as etoposide (DNA damage) or staurosporine (nonselective protein kinase inhibitor) (Fig. 1*D*), suggesting that the increase in miR-593-5p is not a general response associated with cell death. To extend our investigation, we examined differentiated SH-SY5Y cells since the above results were obtained in undifferentiated cells. After 18 days of differentiation (Fig. S2), neurons were exposed to 0.5 mM MPP^+^ for 12 h, and we found a roughly 9-fold increase in miR-593-5p levels (Fig. 1*E*). We further confirmed these results in differentiated neurons derived from human ReNcell VM neural progenitor cells, which represent mesencephalic dopaminergic neurons (13). We treated these neurons with 0.5 mM MPP^+^ for 12 h and observed a roughly 12-fold increase in miR-593-5p levels (Fig. 1*E*). Additionally, we investigated this finding in iPSC-differentiated dopaminergic neurons. Following 30 days of differentiation (Fig. S3), the neurons were exposed to 0.5 mM MPP^+^ for 12 h, and we detected a roughly 7-fold increase in miR-593-5p levels (Fig. 1*E*). Based on the information available in miRbase release 22 (14), it appears that miR-593-5p expression is unique to humans. Our experimental results support this finding, as we were unable to detect the expression of miR-593-5p in primary mouse cortical neurons, even when exposed to MPP^+^ (Fig. S4).

### miR-593-5p contributes to cell death

**Figure 2:**
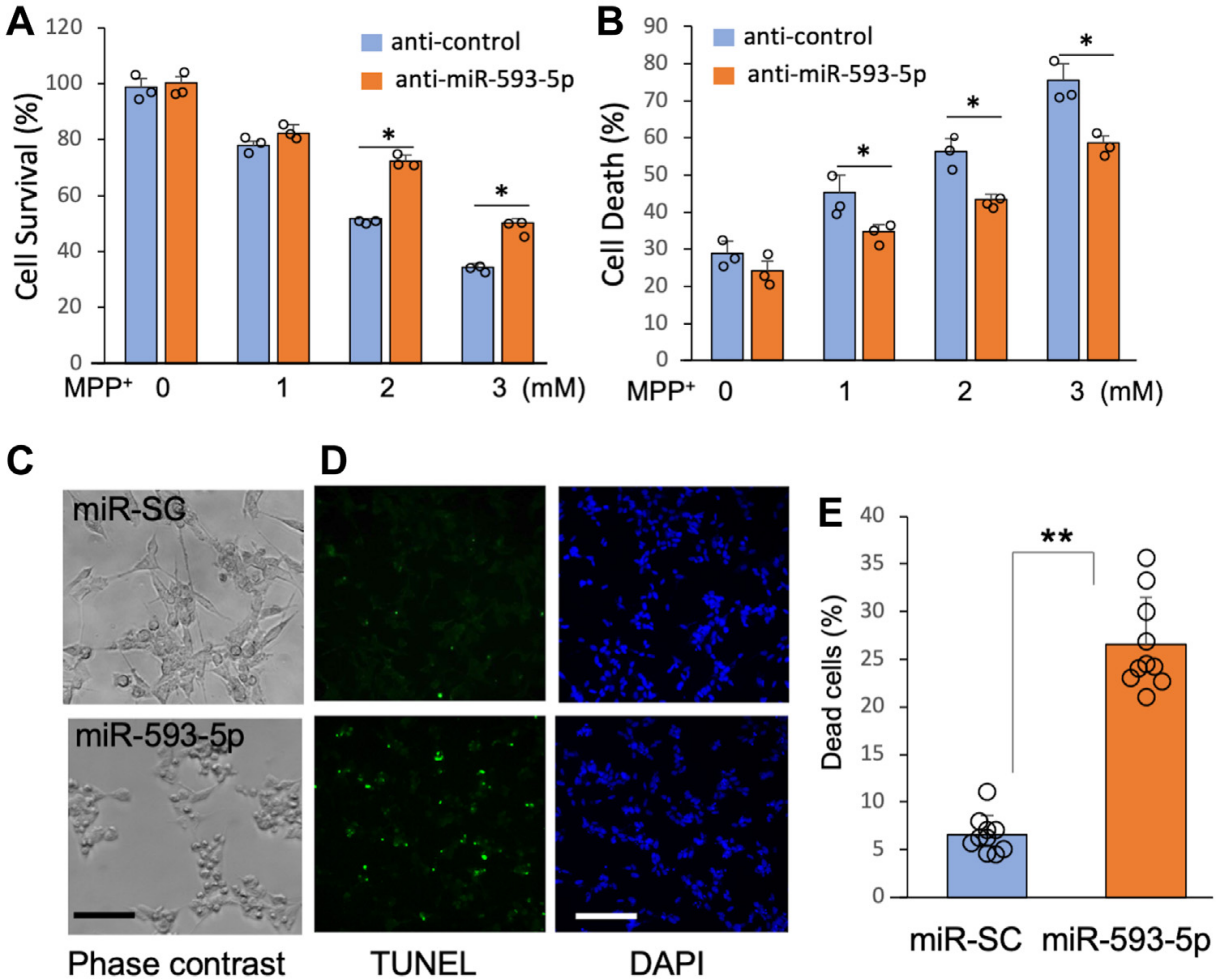
**Effects of miR-593-5p on cell death.***A* and *B*, SH-SY5Y cells were transfected with miR-593-5p inhibitor (anti-miR-593-5p) or control (anti-miR-SC) at a final concentration of 50 nM and treated with MPP^+^ for 24 h. Data are mean ± SD for three biological replicates. Each *circle* represents mean of three technical replicates. *A*, effect of anti-miR-593-5p on cell viability following MPP^+^ was examined using the MTT assay (Promega). Cell viability is expressed as a percentage of untreated anti-control cells (100%). *B*, effect of anti-miR-593-5p on cell death following MPP^+^ was measured by LDH assay (Roche). *C*, cell death was observed using phase contrast microscopy after transfecting miR-593-5p for 48 h in SH-SY5Y. *D*, cell death was analyzed by TUNEL staining after transfecting miR-593-5p for 48 h in SH-SY5Y. The results shown in (*C*) and (*D*) are representative of separate experiments. *E*, the percentage of dead cells (as indicated by *green* fluorescence) was determined in ten different microscopic fields, with a cell (DAPI) count of 20 to 100 per field. Each data point is represented by a *circle*. Data are mean ± SD. *t* test ∗*p* < 0.05, ∗∗*p* < 0.01, ns, nonsignificant. Scale bar represents 40 μm. DAPI, 4′,6′-diamidino-2-phenylindole dihydrochloride; LDH, lactate dehydrogenase; MPP^+^, 1-methyl-4-phenylpyridinium; TUNEL, TdT-mediated-dUTP-X nick end labeling.

Next, we determined the role of miR-593-5p in MPP^+^-induced cell death. Since miR-593-5p is dramatically induced by MPP^+^, we examined if blocking miR-593-5p activity would protect cells. Indeed, the transfection of miR-593-5p inhibitor (anti-miR-593-5p, Thermo Fisher Scientific) resulted in significant protection of cell death, as demonstrated by both cell viability (Fig. 2*A*) and lactate dehydrogenase assays (Fig. 2*B*) in SH-SY5Y cells 24 h after exposure to MPP^+^. Additionally, we found that miR-593-5p levels still increased at 24 h after MPP^+^ exposure, when cell death was measured (Fig. S5). Other miRs, such as miR-708-5p and miR-215-5p, which were hardly affected in miR profiling studies (Table S1), remained unchanged at both 12 h and 24 h after MPP^+^ exposure (Fig. S5). This suggests that the effect of MPP^+^ on miR-593-5p is specific. In addition, overexpression of miR-593-5p by transfecting cells with miR-593-5p mimic (Ambion) led to significant cell death in SH-SY5Y as shown by severe cell shrinkage (Fig. 2*C*) and TdT-mediated-dUTP-X nick end labeling (TUNEL) assay (*In Situ* Cell Death Detection Kit, (Fluorescein), Roche) (Fig. 2, *D* and *E*). Therefore, we suggest that miR-593-5p is toxic to neuronal cells.

### Increased cytosolic Ca^2+^ concentration mediates MPP^+^-induced miR-593-5p expression

**Figure 3:**
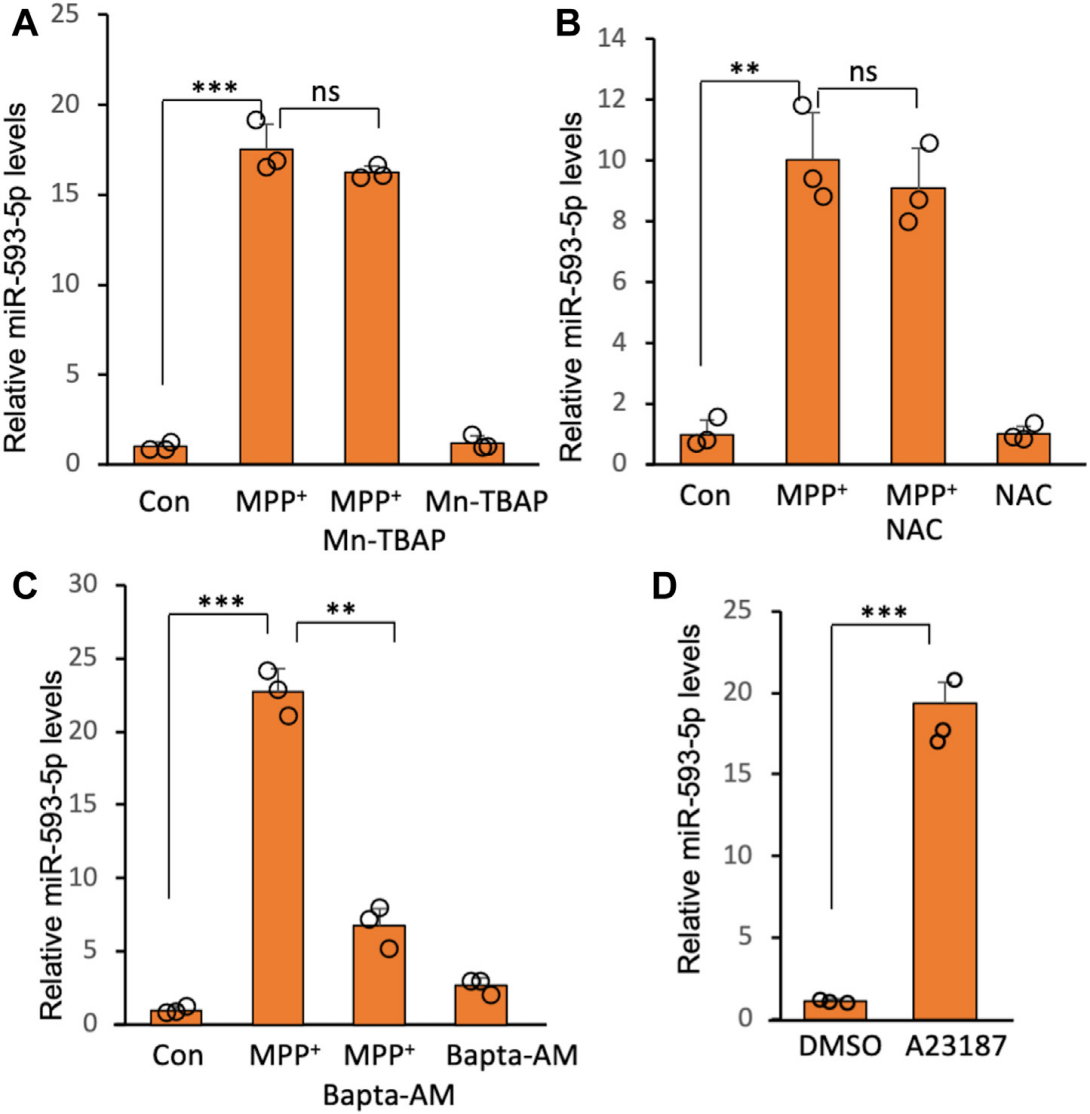
**Intracellular Ca**^**2+**^**mediates miR-593-5p increase following MPP**^**+**^**exposure.** The effect of (*A*) MnTBAP (100 μM), (*B*) NAC (10 mM), or (*C*) BAPTA-AM (40 μM) on MPP^+^-induced miR-593-5p expression were studied. These chemicals were cotreated with MPP^+^ (2 mM) for 12 h. *D*, treatment of calcium ionophore, A23187 (1 μM, 12 h), increases miR-593-5p levels in SH-SY5Y. Data are mean ± SD for three biological replicates. Each *circle* represents the mean of three technical replicates. *t* test, ∗∗*p* < 0.01, ∗∗∗*p* < 0.001, ns, nonsignificant. MnTBAP, Mn(III) tetrakis(4-benzoic acid) porphyrin; MPP^+^, 1-methyl-4-phenylpyridinium; NAC, N-acetylcysteine.

Given that MPP^+^ generates reactive oxygen species (ROS) *via* inhibition of mitochondrial complex I (15), we sought to determine if ROS is involved in MPP^+^-induced miR-593-5p expression. However, neither Mn(III) tetrakis(4-benzoic acid) porphyrin (a superoxide dismutase mimetic) (Fig. 3*A*) nor N-acetylcysteine (a GSH precursor) (Fig. 3*B*) were able to reduce miR-593-5p levels following MPP^+^ treatment, indicating that miR-593-5p expression is not dependent on ROS. In addition to ROS, it has been reported that increased cytoplasmic Ca^2+^ concentrations mediate MPP^+^-induced cell death (16). Interestingly, inhibition of Ca^2+^ by the addition of BAPTA-AM, a cell-permeable intracellular Ca^2+^ chelator, alleviated the MPP^+^-induced increase in miR-593-5p (Fig. 3*C*). Also, there was significant cell death protection under these same conditions (Fig. S6), consistent with previous reports (16). In addition, treatment of A23187, a calcium ionophore, increased miR-593-5p levels (Fig. 3*D*). Therefore, these results suggest that MPP^+^-induced intracellular Ca^2+^ increase leads to the accumulation of miR-593-5p and subsequently mediates cell death.

### MPP^+^-induced increase of miR-593-5p is not mediated by enhanced transcription or by miR processing

**Figure 4:**
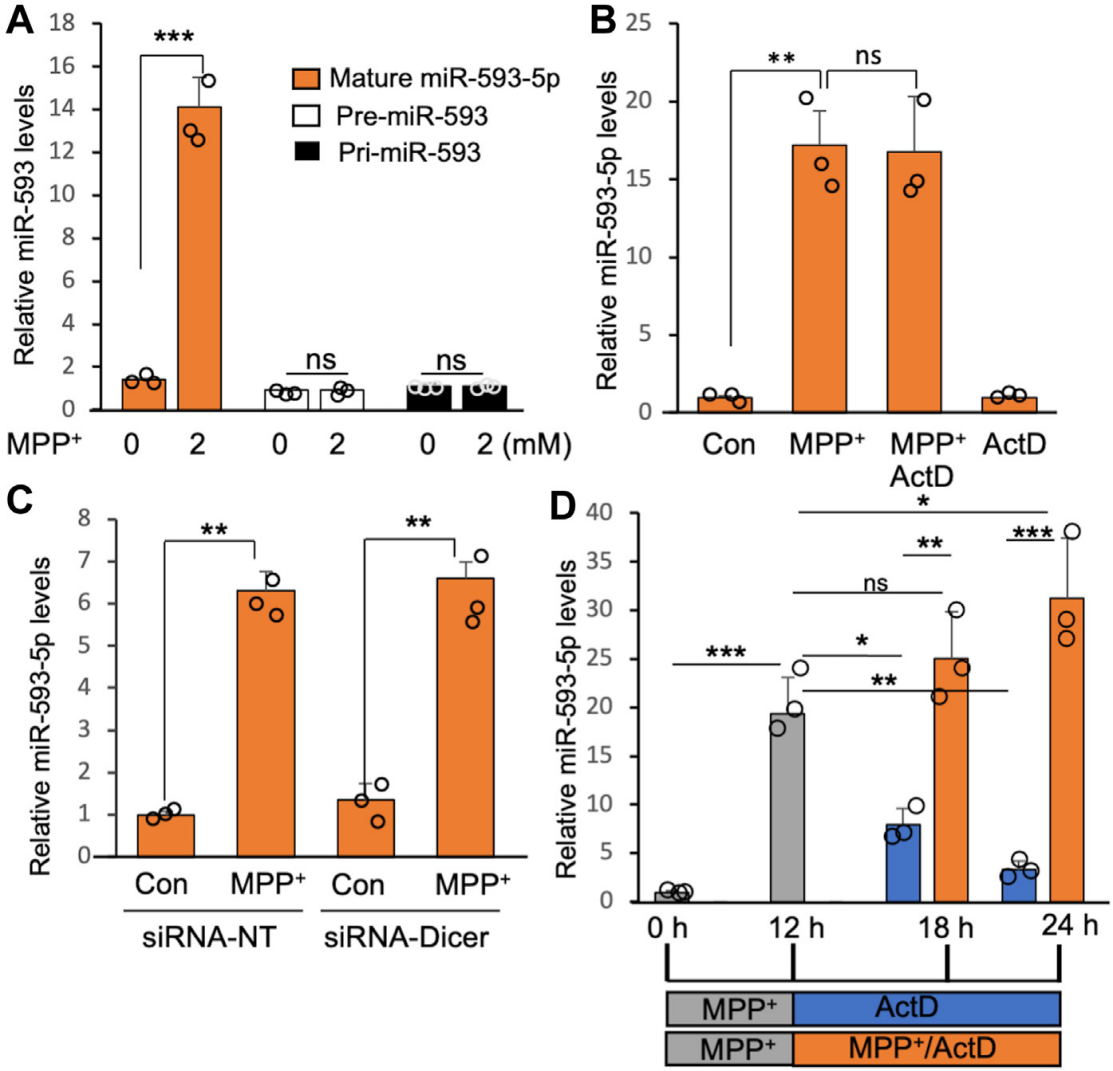
**MPP**^**+**^**-induced increase of miR-593-5p is not mediated by increased transcription and miR processing.***A*, levels of pri-miR-593, pre-miR-593, and miR-593-5p were determined in SH-SY5Y cells after MPP^+^ (2 mM) for 12 h. *B*, the effect of cotreatment with ActD (1 μg/ml) on MPP^+^-induced miR-593-5p expression was examined 12 h after treatment. *C*, effect of Dicer on MPP^+^-induced miR-593-5p expression was investigated after knockdown expression of Dicer by siRNA-Dicer (50 nM) for 48 h, followed by MPP^+^ exposure for 12 h. *D*, treatment with MPP^+^ enhances the stability of miR-593-5p. Following an initial 12-h treatment with MPP^+^ (2 mM), cells were washed and subjected to either ActD (*blue*) or ActD/MPP^+^ (*orange*) treatment for an additional 12 h. Data are mean ± SD for three biological replicates. Each *circle* represents mean of three technical replicates. *t* test (*A*–*C*) and ANOVA with post hoc Turkey (*D*) were performed. ∗*p* < 0.05, ∗∗*p* < 0.01, ∗∗∗*p* < 0.001, ns, nonsignificant. ActD, actinomycin D; MPP^+^, 1-methyl-4-phenylpyridinium; pre-miR, precursor miR; pri-miR, primary miR.

To investigate the mechanism behind the accumulation of miR-593-5p following MPP^+^ exposure, we examined the levels of its precursor forms, pri-miR-593 and pre-miR-593, using quantitative real-time polymerase chain reaction (qRT-PCR). Our results indicated that MPP^+^ exposure did not increase the levels of these precursor forms, while miR-593-5p levels did increase (Fig. 4*A*). Additionally, treatment with actinomycin D (ActD), an inhibitor of transcription, did not decrease the levels of MPP^+^-induced miR-593-5p (Fig. 4*B*), suggesting that transcription is not responsible for the increase. We also investigated whether the increase of miR-593-5p is due to enhanced processing from pre-miR-593 by silencing the RNase, Dicer involved in this process. However, our results showed that MPP^+^-induced miR-593-5p levels were not affected by Dicer knockdown (Fig. 4*C*), indicating that enhanced processing is not responsible for the increase. Moreover, if the increase in miR-593-5p was due to enhanced processing, we would have expected a decrease in pre-miR-593 levels after MPP^+^ exposure, which was not observed (Fig. 4*A*). Successful knockdown of Dicer expression was confirmed by qRT-PCR (Fig. S7). Since transcription and processing do not seem to be responsible for the increase in miR-593-5p, we investigated whether the increased stability of the mature miR could account for this increase. To this end, we exposed cells to MPP^+^ for 12 h to induce miR-593-5p expression and then washed the cells and treated them with either ActD or a combination of MPP^+^ and ActD for an additional 12 h, as depicted in Figure 4*D*. Our results showed that miR-593-5p levels decreased in the ActD-treated sample but remained stable (or even increased) in the MPP^+^/ActD-treated sample, suggesting that MPP^+^ exposure increases the stability of miR-593-5p (Fig. 4*D*). Therefore, we conclude that the accumulation of miR-593-5p following MPP^+^ exposure is most likely due to increased stability of the mature miR rather than enhanced transcription or processing.

### miR-593-5p inhibits PINK1/Parkin-mitophagy pathway

**Figure 5:**
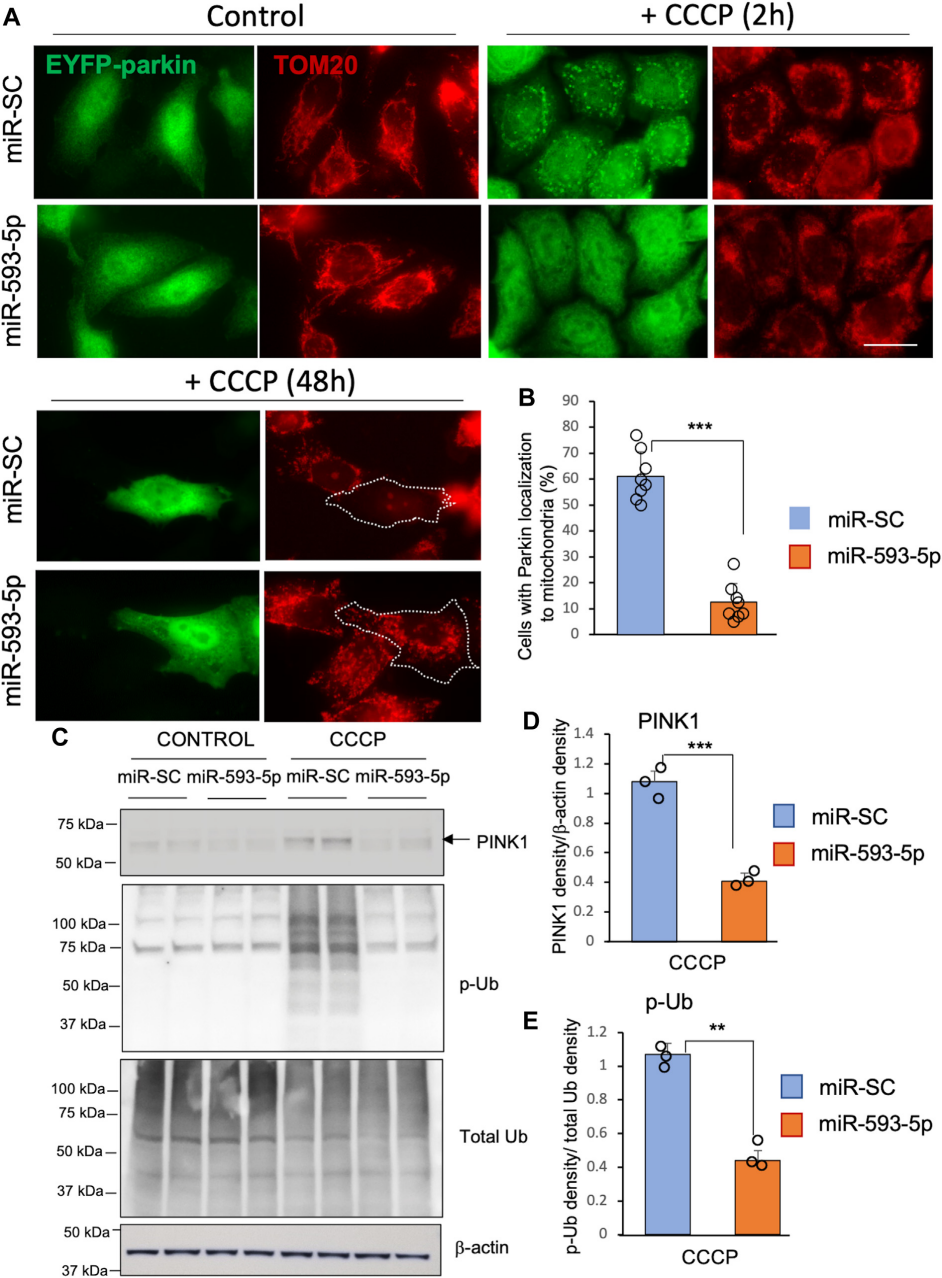
**miR-593-5p inhibits the pathway mediated by PINK1 and Parkin.***A*, inhibition of mitochondrial localization of Parkin and mitochondria clearance by miR-593-5p. HeLa cells were cotransfected with miR-593-5p or miR-SC (50 nM) along with EYFP-Parkin (Addgene) for 24 h and treated with CCCP (10 μM) for 2 h and 48 h. Mitochondria was stained with anti-TOM20 (Santa Cruz, sc-17764). *B*, the percentage of cells with EYFP-Parkin localization to mitochondria was determined in eight different microscopic fields, with a cell count of 20 to 60 per field. Each data point is represented by a *circle*. Data are mean ± SD. *t* test, ∗∗∗*p* < 0.001. *C*, SH-SY5Y cells were transfected as indicated for 24 h and treated with CCCP (10 μM) for 2 h. Western blots were performed with cell lysates using anti-PINK1 (Novus biologicals, BC100-494), anti-p-Ub (Milipore, #ABS1513-I), anti-Ub (Santa Cruz, P4D1), and anti-β-actin (Sigma, AC-74). *D*, band densities of PINK1 in Western blot were measured and normalized to those of β-actin. *E*, band densities of p-Ub in Western blot were measured and normalized to those of total Ub. Band densities were calculated from three independent Western blot experiments. Data (*D* and *E*) are mean ± SD for three biological replicates. Each *circle* represents the mean of two technical replicates. *t* test, ∗∗*p* < 0.01, ∗∗∗*p* < 0.001. Scale bar represents 20 μm. CCCP, carbonyl cyanide m-chlorophenylhydrazone; PINK1, PTEN-induced putative kinase 1; p-Ub, phospho-ubiquitin.

As miR-593-5p is highly upregulated by the mitochondrial toxin MPP^+^, we sought to explore the effect of miR-593-5p on mitochondria. Mitophagy, a process that degrades dysfunctional and excess mitochondria, is mediated by Parkin and is critical for maintaining mitochondrial health. Parkin is recruited from the cytosol to mitochondria *via* PINK1 kinase activity (17, 18, 19). Failure to eliminate dysfunctional mitochondria due to *parkin* gene mutation or *pink1* gene mutations has been implicated in PD pathogenesis (20). To explore the role of miR-593-5p in mitophagy, we first examined the mitochondrial localization of Parkin in HeLa cells after depolarizing mitochondria with the mitochondrial uncoupler carbonyl cyanide m-chlorophenylhydrazone (CCCP) for 2 h. We found that while EYFP-Parkin localized to mitochondria after CCCP exposure in cells transfected with a control miR (miR-SC), consistent with previous findings (21), the mitochondrial localization of Parkin was dramatically reduced by about 80% upon transfection of miR-593-5p (Fig. 5, *A* and *B*). In addition, miR-593-5p expression significantly inhibited the mitochondrial localization of Parkin following CCCP in SH-SY5Y cells (Fig. S8, *A* and *B*). Furthermore, transfection of pre-miR-SC in EYFP-Parkin–expressing cells resulted in a significant decrease in the mitochondrial mass (as shown in TOM20 staining) 48 h after the addition of CCCP (Fig. 5*A*). However, the expression of miR-593-5p did not reduce the mitochondrial mass in EYFP-Parkin–expressing cells (Fig. 5*A*).

We then investigated whether miR-593-5p attenuates PINK1 accumulation, as PINK1 is critical for Parkin translocation to damaged mitochondria and subsequent mitophagy (17, 18, 19). Consistent with previous reports (18, 19), we observed that endogenous full-length PINK1 (65 kDa) accumulates following 2 h exposure of CCCP in SH-SY5Y cells (Fig. 5*C*). However, the transfection of miR-593-5p significantly decreased the accumulation of PINK1 by about 60% after CCCP treatment (Fig. 5, *C* and *D*). PINK1 is known to activate mitophagy by phosphorylating ubiquitin at serine 65 (22, 23). Therefore, we also evaluated the levels of phospho-ubiquitin (p-Ub). Our data reveal that miR-593-5p overexpression resulted in approximately 56% decrease in CCCP-induced p-Ub, while the overall levels of ubiquitin remained unchanged (Fig. 5, *C* and *E*). These findings indicate that miR-593-5p functions to inhibit the pathway mediated by PINK1 and Parkin, which play a critical role in mitophagy.

### miR-593-5p targets the coding sequence (CDS) of PINK1 mRNA

**Figure 6:**
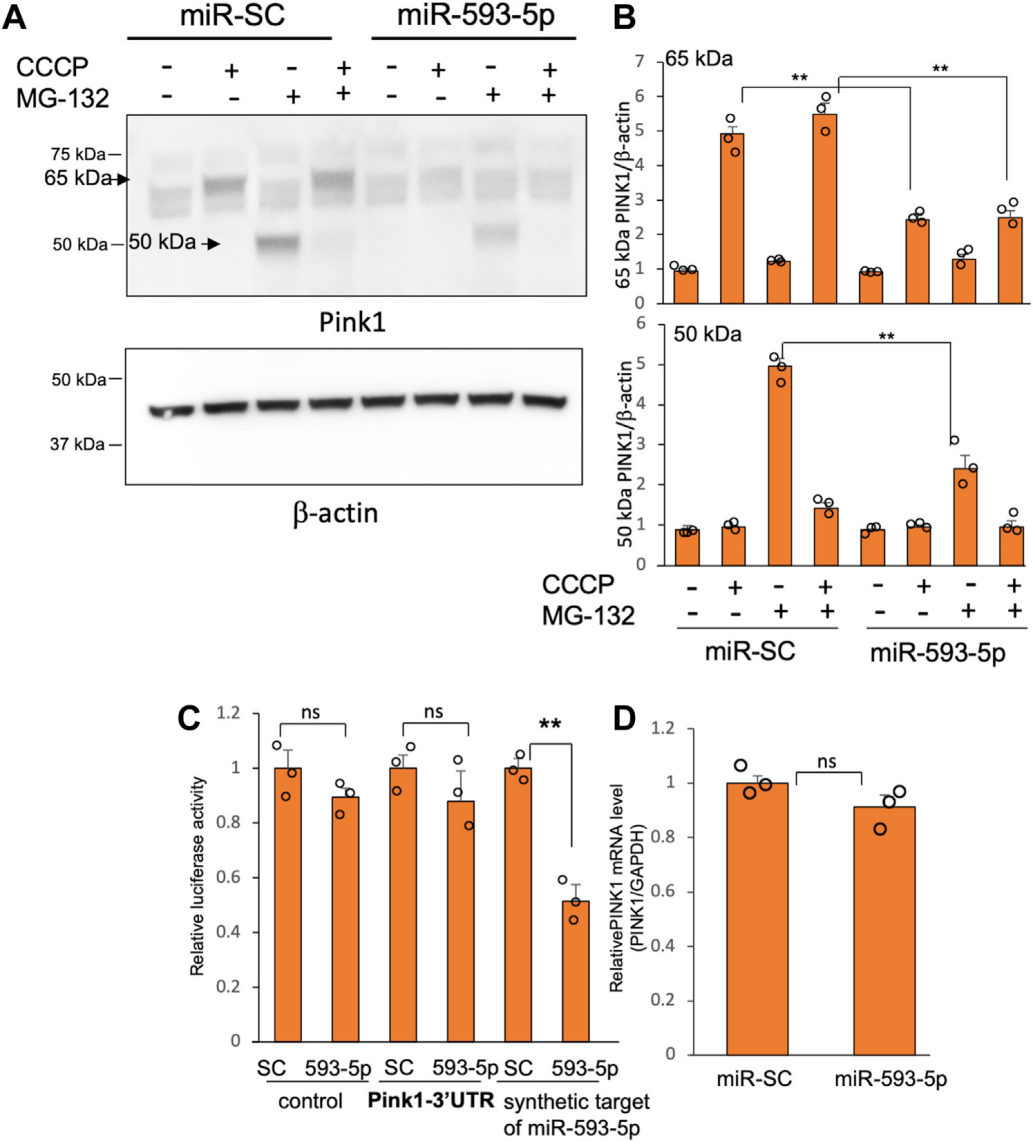
**miR-593-5p directly targets PINK1 gene.***A*, SH-SY5Y transfected with miR-593-5p or miR-SC were treated with CCCP (10 μM), MG-132 (10 μM), or both for 2 h. Western blots were performed using Pink1 and β-actin antibodies. *B*, the band intensities of 65 kDa and 50 kDa Pink1 were measured from three independent Western blot experiments and normalized to those of β-actin. Statistical significance was determined by ANOVA followed by Bonferroni’s post hoc test. Data are mean ± SD for three biological replicates. *t* test, ∗∗*p* < 0.01. *C*, reporter assay using luciferase construct containing PINK1-3′UTR or synthetic miR-593-5p′s target sequence. SH-SY5Y cells were cotransfected as indicated along with pSV-β-galactosidase (Promega). Luciferase activity was normalized against β-galactosidase activity. *D*, determination of PINK1 mRNA levels in SH-SY5Y cells transfected with miR-593-5p or miR-SC by qRT-PCR. Data (*C* and *D*) are mean ± SD for three biological replicates. Each *circle* represents the mean of three technical replicates. *t* test, ∗∗*p* < 0.01, ns, nonsignificant. CCCP, carbonyl cyanide m-chlorophenylhydrazone; PINK1, PTEN-induced putative kinase 1.

Loss of PINK1 stability by miR-593-5p in damaged mitochondria may be due to direct targeting of PINK1 mRNA or due to targeting factors related to PINK1 stability. The accumulation of the 52-kDa cleaved form of PINK1 was observed when proteasome was inhibited by MG-132, as previously reported (18, 24). However, when miR-593-5p was overexpressed, it prevented the accumulation of the 52-kDa cleaved form of PINK1 to a similar extent as the 65-kDa full-length form of PINK1 (Fig. 6, *A* and *B*). This result suggests that miR-593-5p directly targets PINK1 mRNA and thus reduces PINK1 expression. Therefore, we sought to determine whether miR-593-5p targets the 3′-UTR of PINK1 mRNA. Overexpression of miR-593-5p did not decrease the luciferase activity from cells transfected with a PINK1-3′-UTR luciferase construct, while it decreased the luciferase activity from a luciferase construct containing a synthetic target sequence of miR-593-5p (Fig. 6*C*). Additionally, we found that overexpression of miR-593-5p had little effect on the levels of PINK1 mRNA (Fig. 6*D*).

**Figure 7:**
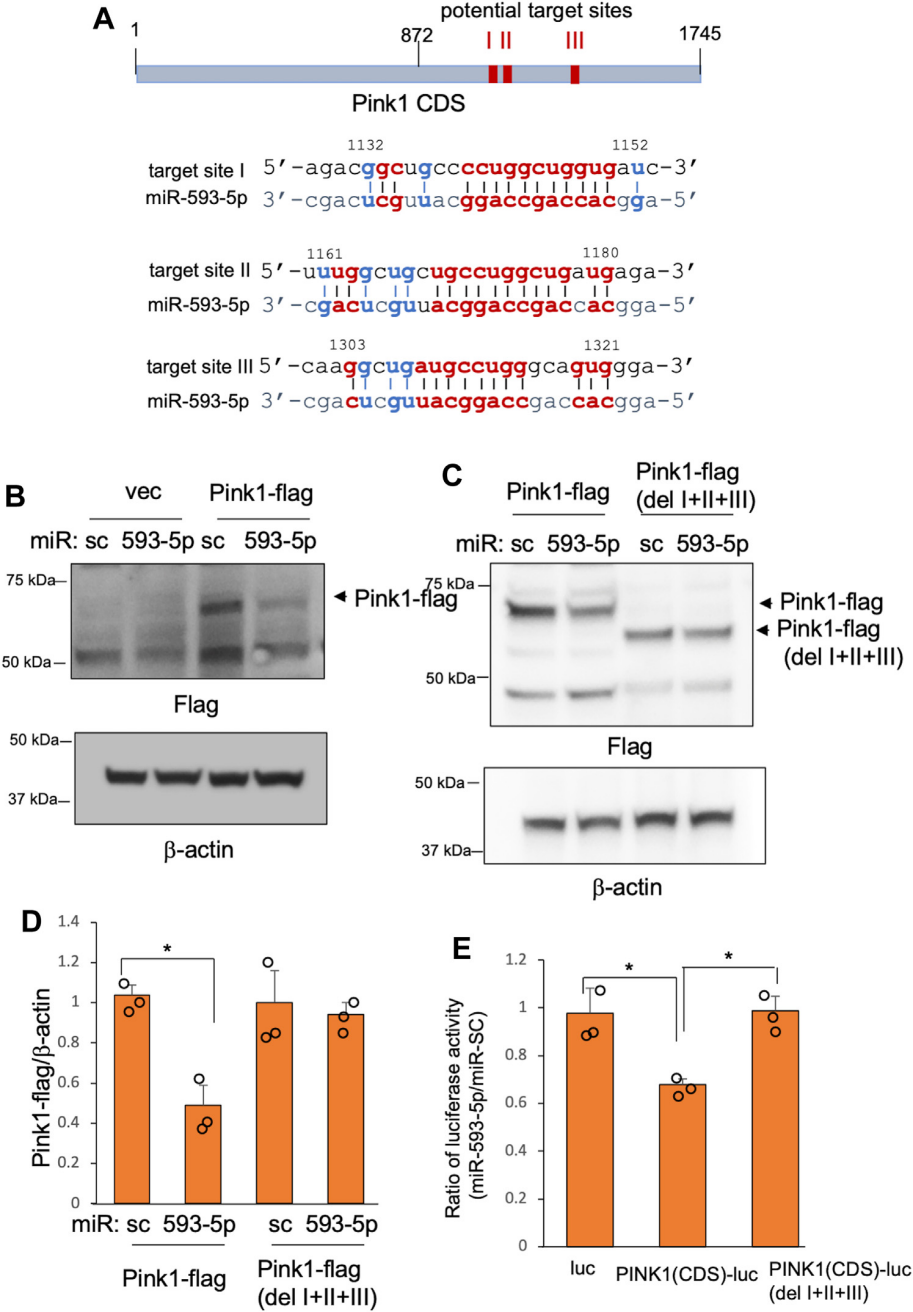
**miR-593-5p targets the CDS of PINK1 mRNA.***A*, schematic drawings illustrating the potential target sites of miR-593-5p in the CDS of PINK1 mRNA and potential base-pairings between miR-593-5p and target sites (I, II, and III). Watson-Crick base pairs are represented in *red* and wobble base pairs (G:U) are represented in *blue*. *B* and *C*, SH-SY5Y cells were transfected as indicated. Western blots were performed with anti-FLAG and anti-β-actin. *D*, band densities of PINK1-flag were measured and normalized to those of β-actin. Band densities were calculated from three independent Western blot experiments of *C*. Data are mean ± SD for three biological replicates. *E*, SH-SY5Y cells were transfected as indicated along with pSV-β-galactosidase. Luciferase activities were measured and normalized to β-galactosidase activity. Data are mean ± SD for three biological replicates. Each *circle* represents the mean of three technical replicates. *t* test, ∗*p* < 0.05. CDS, coding sequence; PINK1, PTEN-induced putative kinase 1.

There is growing evidence that miRs can target genes through CDS and 5′-UTR in addition to 3′-UTR sequences (25, 26). Sequence analysis identified three potential target sites for miR-593-5p in the CDS of PINK1 mRNA (Fig. 7*A*). These target sequences are noncanonical as they lack the typical seed base pairing with miR-593-5p but instead show extensive base-pairing across the entire sequence of miR-593-5p. To determine whether miR-593-5p targets the CDS of PINK1 mRNA, we transfected a PINK1 expression plasmid containing only the CDS, which was tagged with FLAG in the C-terminal, with miR-593-5p. As shown in Figure 7, *B*–*D*, miR-593-5p reduced the expression of PINK1-FLAG, but not of PINK1-FLAG with a deleted 195 bp DNA region containing three potential target sites. Further, miR-593-5p reduced luciferase activity in constructs in which PINK1 CDS was fused to the firefly luciferase gene, but not in constructs without PINK1 CDS or PINK1 CDS lacking three potential target sites (Fig. 7*E*). Taken together, these results suggest that miR-593-5p reduces PINK1 expression by inhibiting translation through directly targeting the CDS of PINK1 mRNA.

**Figure 8:**
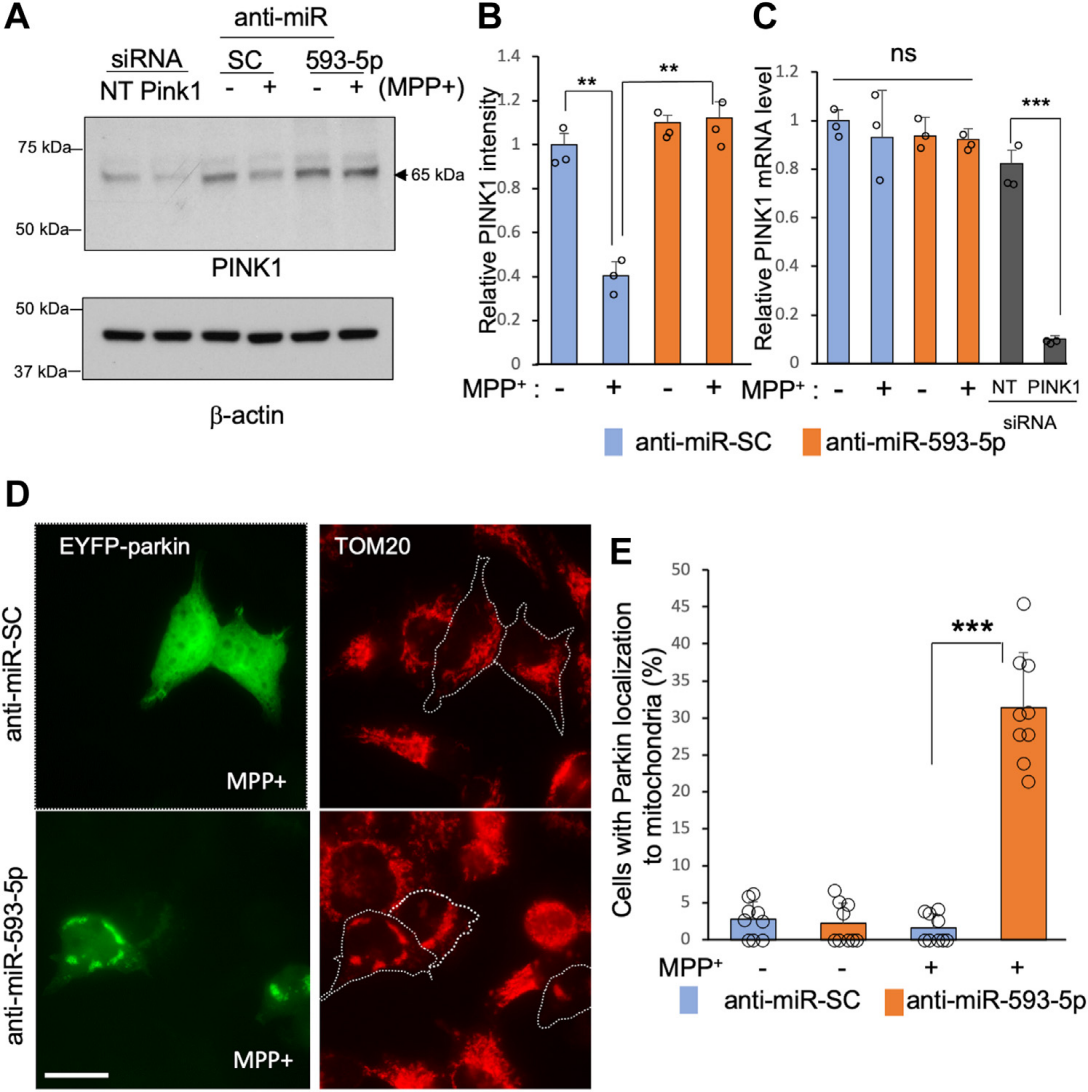
**Decrease in PINK1 expression after MPP**^**+**^**exposure is mediated by increased miR-593-5p in SH-SY5Y.***A*, SH-SY5Y cells were transfected with anti-miR-593-5p (50 nM) or anti-miR-SC (50 nM) for 24 h and then exposed to MPP^+^ (2 mM) for 24 h. In addition, siRNA-PINK1 (50 nM) and siRNA-NT (50 nM) were transfected as controls. *B*, PINK1 band intensities were measured from the three separate experiments and normalized to β-actin densities. Data are mean ± SD for three biological replicates. *C*, PINK1 mRNA levels were determined in the same experimental samples as (*A*), and their levels were normalized to GAPDH mRNA levels. Data are mean ± SD for three biological replicates. Each *circle* represents the mean of three technical replicates. *D*, SH-SY5Y cells transfected with EYFP-parkin were transfected with anti-miR-593-5p (50 nM) or anti-miR-SC (50 nM) for 24 h and then exposed to MPP^+^ for 24 h. Mitochondria were stained with anti-TOM20. *E*, the percentage of cells with EYFP-Parkin localization to mitochondria was determined in nine different microscopic fields, with a cell count of 10 to 50 per field. Each data point is represented by a *circle*. Data are mean ± SD. *t* test, ∗∗*p* < 0.01, ∗∗∗*p* < 0.001. Scale bar represents 20 μm. MPP^+^, 1-methyl-4-phenylpyridinium; PINK1, PTEN-induced putative kinase 1.

Next, we investigated the effect of MPP^+^-induced miR-593-5p increase on the mitophagy pathway in SH-SY5Y by transfecting cells with control or anti-miR-593-5p before treatment with MPP^+^. Results showed that MPP^+^ treatment reduced PINK1 expression in cells transfected with control anti-miR-SC, but not in cells transfected with anti-miR-593-5p (Fig. 8, *A* and *B*). Furthermore, the decrease in PINK1 expression in cells transfected with control anti-miR-SC was not caused by a decrease in PINK1 mRNA levels (Fig. 8*C*). This suggests that MPP^+^-induced miR-593-5p inhibits PINK1 translation. Then, we aimed to investigate the localization of Parkin in mitochondria in response to MPP^+^ treatment. To enhance visualization, we transfected EYFP-Parkin into SH-SY5Y cells. We found that when exposed to MPP^+^, EYFP-Parkin did not move to mitochondria in SH-SY5Y cells transfected with anti-miR-SC, whereas inhibition of miR-593-5p led to mitochondrial localization of EYFP-Parkin in about 30% of cells (Fig. 8, *D* and *E*). In addition, we found that MPP^+^ exposure led to a decrease in p-Ub levels in cells transfected with control anti-miR-SC, but not in cells transfected with anti-miR-593-5p (Fig. S9). Therefore, these results suggest that miR-593-5p accumulation after MPP^+^ exposure leads to a decrease in PINK1 levels, p-Ub levels, and mitochondrial localization of Parkin.

## Discussion

MiR-593-5p is found and expressed only in humans, according to the official miR database, miRbase release 22 (14). In general, the levels of specific human miRs remain very low in the human brain (27), because it is thought that high levels of evolutionarily recently emerged miRs can regulate the expression of numerous genes and thus potentially lead to deleterious repression of essential genes (28). Some stressful conditions likely lead to derepression of newly emerged miRs, which subsequently results in the demise of cells and the development of disease. Although these human-specific miRs can target the same mRNAs/pathways that other evolutionarily conserved miRs can also target, human-specific miRs may mediate the impact of genetic/environmental factors on specific cellular pathways. This link may not exist in nonhuman species, thereby underlying the susceptibility of humans to certain diseases and environmental challenges. For example, while MPTP/MPP^+^ can kill murine dopaminergic neurons, mice lack miR-593-5p expression and are known to be relatively resistant to MPTP compared to humans (29). We hypothesize that a possible mechanism for this resistance is in part due to the deficiency of miR-593-5p in mouse cells. Thus, increased expression of miR-593-5p exacerbates the susceptibility of human cells to the same stress compared to mouse cells. Current animal models of neurodegenerative diseases such as PD do not replicate the true pathophysiology of the human disease, so results from animal models are often not translatable to the clinical situation. Elucidating human-specific factors involved in neuropathogenesis including human-specific miRs, such as miR-593-5p, may help bridge the gap.

Dysregulation of cytosolic calcium (Ca^2+^) has been linked to the development of neurodegenerative disorders, such as PD (30, 31, 32). In addition, several studies have shown that MPP^+^ can induce cell death in neuronal cells through Ca^2+^-dependent mechanism (16, 33). In line with this, our studies have also revealed that cytosolic Ca^2+^ mediates MPP^+^-induced miR-593-5p accumulation. Further, a series of experiments including measurement of pre- and pri-miR-593 levels and measurement of miR-593-5p with inclusion of Act D or knockdown of Dicer expression showed that MPP^+^-induced miR-593-5p accumulation was not dependent on increase of transcription and miR processing. This suggests that miR-593-5p accumulation is due to increased stability (decreased degradation). Taken together, we suggest that MPP^+^-induced cytosolic Ca^2+^ decreases the degradation of miR-593-5p, resulting in the accumulation of miR-593-5p.

Despite a detailed understanding of miR biogenesis, the mechanisms of miR turnover remain largely unknown. However, miR stability appears to be highly regulated depending on specific miRs and cellular contexts (34, 35, 36). For example, Tudor-SN, also known as staphylococcal nuclease and tudor domain containing 1 (SND1), has been reported to cleave and downregulate specific miRs such as miR-31-5p, miR-29b-3p, and miR-125a-5p without significantly altering the levels of pre- or pri-miRs (37). In particular, the miR-593-5p gene is embedded in the intronic region of the SND1 gene (38). Since intragenic miRs are known to be functionally coregulated and coexpressed with host genes, it will be interesting to investigate whether SND1 is involved in the regulation of miR-593-5p stability. Another mechanism for turnover is controlled by highly complementary target RNAs that, when bound to cognate miRNAs, result in miRNA decay instead of target inhibition *via* a process known as target-directed miRNA degradation (TDMD) (39, 40, 41, 42). If TDMD contributes to the degradation of miR-593-5p, the target RNA mediating TDMD of miR-593-5p is expected to decrease in a Ca^2+^-dependent manner after exposure to MPP^+^, resulting in the accumulation of miR-593-5p. Identifying the target RNA will help elucidate the potential TDMD mechanism by which miR-593-5p is degraded.

We found that miR-593-5p targeted the CDS of PINK1 mRNA and decreased PINK1 expression without altering PINK1 mRNA levels. Although most studies have shown that miRs target the 3′-UTR of the target mRNA, targeting the CDS is not uncommon. In fact, cross-linking and immunoprecipitation studies of human cells mapping mRNA fragments bound by miRs provided genome-wide evidence that miR binding is as frequent in CDS as in 3′-UTRs (25, 26). Although it is well established that miRs destabilize target mRNAs by binding to 3′-UTRs, several studies have shown that miR binding to CDS inhibits translation (43, 44, 45), which is consistent with our data. What is the regulatory advantage of CDS targeting over 3′-UTR targeting? One potential advantage of CDS targeting over 3′-UTR targeting is that it allows for faster regulation of protein levels. Previous studies have shown that translation inhibition by CDS targeting occurs before mRNAs are deadenylated by 3′-UTR targeting (43), which involves mRNA deadenylation and subsequent degradation. In healthy conditions, PINK1 protein is transported to mitochondria, cleaved, and degraded by the proteasome. When cells are stressed, PINK1 accumulates in mitochondria and triggers PINK1/Parkin-mediated autophagy to eliminate dysfunctional mitochondria and promote cell survival. However, when stress persists, cells are doomed to die by the accumulated miR-593-5p. In this situation where the cytoprotective response, such as PINK1/Parkin-mediated mitophagy, still operates, miR-593-5p can speed up the cell death process by targeting the CDS of PINK1 mRNA.

We discovered that MPP^+^ treatment led to a decrease in PINK1 expression, but transfection with anti-miR-593-5p under the same conditions did not reduce PINK1 expression, thereby protecting against cell death after MPP^+^ treatment. This is consistent with previous research that showed PINK1 expression decreased after MPP^+^ treatment, without affecting PINK1 mRNA levels in SH-SY5Y cells (46). In this study, PINK1 was shown to be degraded by Bcl2-associated athanogene 6 (BAG6), a protein that is highly elevated after MPP^+^ treatment. This suggests that cells use multiple ways to inhibit PINK1-mediated mitophagy, leading to cell death after exposure to MPP^+^. Inhibiting these pathways may lead to the development of therapeutic interventions for neurodegenerative disorders. Since miR-593-5p has low basal levels, blocking its expression is unlikely to have unwanted side effects, making it an attractive target for therapeutic approaches.

## Experimental procedures

### Materials

MPP^+^, etoposide, CCCP, and actinomycin D were purchased from Sigma-Aldrich. All siRNAs used in this study are in the form of dicer-substrate siRNA purchased from Integrated DNA Technologies. Scrambled miR control (miR-SC), miR-593-5p, anti-miR negative control (anti-control), and anti-miR-593-5p were purchased from Thermo Fisher Scientific. Human miRNome miScript miRNA PCR Array and miScript primer assays for measuring miR levels such as miR-593-5p, miR-593-3p, miR-708-5p, miR-215-5p, and U6 were purchased from Qiagen.

### Plasmids

EYFP-Parkin was obtained from Addgene. The PINK1-FLAG plasmid was constructed using pcDNA-3.1-FLAG in which DNA sequence encoding the FLAG sequence was preinserted between XhoI and XbaI of pcDNA-3.1. Human PINK1 CDS was amplified from pEYFP-N1-PINK1 (Addgene) using the following primers: 5′-CGGAAGCTTATGGCGGTGCGACAGGCGCTGGGC-3′ and 5′-GGC CTCGAGCAGGGCTGCCCTCCATGAGCAGAG-3′. The PCR product was inserted into pcDNA-3.1-FLAG using HindIII and XhoI. The PINK1-luc plasmid expressing PINK1 and luciferase in-frame was constructed from the pGL3-promoter (Promega) using HindIII and NcoI after PINK1 cDNA was amplified using the following primers: 5′-CGGAAGCTTATGGCGGTGCGACAGGCGCTGGGC-3′ and 5′-GAATCATGACCAGGGCTGCCCTCCATGAGCAG-3′. The PINK1-FLAG (Δ1-2-3) with three target sites deleted was constructed using the following primers: 5′-TTGTGGAGCTGGACCCAGACAGCATCGGCCTGCAGTTGCC-3′ and 5′-GGCAACTGCAGGCCGATGCTGTCTGGGTCCAGCTCCACAA-3′. Human PINK1 3′-UTR was amplified from human genomic DNA using the following primers: forward primer: 5′-AAATCTAGATGTCCCTGCATGGAGCTGGT-3′, reverse primer: 5′- AAATCTAGATCAGTTGAAGACAACCTTTA-3′. The PCR product was inserted into pGL4.51 plasmid (Promega) using Xba1 restriction enzyme in the correct orientation. The firefly luciferase reporter construct containing the target sequence of miR-593-5p was prepared using two oligonucleotides: Top strand 5′-CTAGAGCTGAGCAATGCCTGGCTGGTGCCT-3′ and bottom strand 5′-CTAGAGGCACCAGCCAGGCATTGCTCAGCT-3′. These two oligonucleotides were annealed and inserted into pGL4.51 plasmid and the correct orientation was confirmed by DNA sequencing.

### Cell cultures and transfection

Human neuroblastoma cell line, SH-SY5Y was purchased from American Type Culture Collection. Cells were maintained in DMEM (Invitrogen) supplemented with 10% FBS (Invitrogen). Cells were transfected with miRs and siRNAs using Lipofectamine RNAiMax (Invitrogen) according to the manufacturer’s instructions. Transfections of plasmids were performed using Lipofectamine 3000 (Invitrogen) according to the manufacturer’s instructions. Differentiation of SH-SY5Y cells were performed as reported previously (47). The human neural progenitor cell line, ReNcell VM was purchased from Millipore. Maintenance and differentiation to tyrosine hydroxylase–positive dopaminergic neurons were performed as described previously (13). Primary mouse cortical neurons were isolated and cultured as described previously (48). Experiments were performed after 16 days of differentiation. For the differentiation of iPSC, we used the dual-SMAD inhibition method for floor plate–based midbrain DA neuron induction (49, 50). The iPSC line ND41865, which originated from a 64-year-old male normal subject (NDS00159), was obtained from the NINDS Human Cell and Data Repository located at Infinity Biologix Inc. For the iPSC culture and dopaminergic neuron differentiation, the following materials were used. ReLeSR (Stem Cell Technology), mTESR (Stem Cell Technology), Knockout Serum Replacement (Thermo Fisher Scientific), Matrigel (Corning), StemBeads FGF2 (Stem Culture), Neurobasal medium (Life Technologies), L-Glutamine (100×) (Thermo Fisher Scientific), N2 supplement (Gibco Life Technologies), B27 (Life Technologies), Y-27632 (Peprotech), SB431542 (Peprotech), LDN193189 (Peprotech), CHIR99021 (Peprotech), SHH C25II (R&D), Brain-derived neurotrophic factor (Peprotech), Ascorbic acid (Sigma Aldrich), Dibutyryl cAMP (Santa Cruz), Glial cell line-derived neurotrophic factor (Peprotech), Transforming growth factor type β3 (TGFβ3) (Peprotech), DAPT (Peprotech), Poly-L-Ornithine (Sigma Aldrich), Mouse Laminin I (Invitrogen), Fibronectin (FN) (Thermo Fisher Scientific), Accutase (Innovative Cell Technologies).

### Cell viability and death assay

Cell viability was determined using the MTT assay with the CellTiter 96 AQueous Cell Proliferation Assay kit (Promega) following the manufacturer's protocol. Cell death was measured using the Cytotoxicity Detection Kit^PLUS^ (Roche) based on the measurement of lactate dehydrogenase activity released from damaged/dying cells according to manufacturer’s instructions.

### RNA extraction and quantitative real-time PCR

Total RNA was extracted using QIAzol Lysis Reagent (Qiagen). The level of mature miR-593-5p and precursor miR-593 were analyzed with miScript miRNA assay kit (Qiagen) using Applied Biosystems 7500 Real-time PCR system. Relative miR expression level was normalized to U6. Relative miR levels were calculated by the 2^−ΔΔCt^ method. For the analysis of pri-miR-593, we used the following PCR primers: 5′-CTATGCTGGGCAGGATGATTT-3′ and 5-ACCTCTAGAGAAGGAGAAACCC-3′. For the analysis of PINK1 mRNA levels, cDNA was generated by reverse transcription using the Superscript III RT kit (Invitrogen). Quantitative RT-PCR was done using SYBR select mastermix (Life Technologies). The level of GAPDH mRNA was used for normalization control. PCR primer sequences were as follows: human PINK1 (5′-CTGGGCCTCATCGAGGAAAA-3′ and 5′-AGCCCTTACCAATGGACTGC-3′), human GAPDH (5′-GACAGTCAGCCGCATCTTCT-3′ and 5′-GCGCCCAATACGACCAAATC-3′).

### Western blot analysis

Western blot analysis was performed as reported previously (51). Briefly, cells were harvested in PBS containing 2% SDS with protease cocktail and phosphatase inhibitors (Roche Applied Science) and sonicated for 15 s. Protein concentration was measured by BCA Protein Assay Reagent (Thermo Fisher Scientific). Proteins were resolved on a 4 to 15% SDS-PAGE gel (Genscript) and transferred onto PVDF membrane. The following primary antibodies were used: anti-PINK1 (Novus biologicals, BC100-494), anti-phospho-ubiquitin (Milipore, ABS1513-I), anti-ubiquitin (Santa Cruz Biotech., sc-8017), anti-FLAG (Sigma-Aldrich, F1804), and anti-β actin (Sigma-Aldrich, A5316). The following secondary antibodies were used: horseradish peroxidase-conjugated anti-rabbit (R&D Systems, HAF008) or anti-mouse antibody (R&D Systems, HAF007). Band signals were generated with the enhanced chemiluminescence system (Pierce, 34078). Band intensities were measured using Image J (NIH) and normalized to β-actin.

### TUNEL assay

TUNEL assay was performed by *In Situ* cell death detection kit, fluorescein (Roche Applied Science) according to manufacturer’s instruction. In brief, after fixation and permeabilization, cells grown in cover slips were incubated with TUNEL reaction mixture at 37 °C for 1 h in a humidified chamber. Cover slips were then washed in PBS, stained with 4′,6′-diamidino-2-phenylindole dihydrochloride, and mounted. Cover slips were imaged using Leica DMi8 fluorescence microscope imaging system (DMi8M). Number of TUNEL-positive (green) cells were counted in five different microscopic fields including 100 to 200 cells for each sample.

### Immunocytochemistry

Cells were fixed in 4% paraformaldehyde in PBS for 15 min, washed with PBS, and permeabilized with 0.5% Triton X-100 in PBS for 7 min. After washing with PBS and blocking with 5% donkey serum for 20 min, cells were incubated with primary antibodies diluted in 1% donkey serum at room temperature for 1 h. The primary antibodies used in this study were anti-MAP2 (SantaCruz, sc-20172), anti-tubulin βIII (Sigma-Aldrich, MAB5564), anti-tyrosine hydroxylase (Santa Cruz, sc-25269), and anti-Tom20 antibody (Santa Cruz Biotech., sc-17764). After washing with PBS, samples were incubated with rhodamine red-conjugated anti-rabbit IgG (Jackson Immunoresearch, 111-295-003) diluted in PBS containing 1% donkey serum for 1 h at room temperature. For nuclear staining, cells were incubated with 1 μg/ml 4′,6′-diamidino-2-phenylindole dihydrochloride (Sigma-Aldrich) in PBS for 1 min at room temperature. Cells were washed three times with PBS and analyzed under a Leica DMi8 fluorescence microscope system.

### Reporter gene assay

Cells were cotransfected with luciferase reporter constructs and internal control plasmid, pSV-β-galactosidase (Promega) in the presence of miR-593-5p or miR-SC. After cell lysis using Glo Lysis Buffer (Promega), luciferase activity was measured with Steady-Glo Luciferase Assay (Promega) by a Wallac 1420 Multilabel Counter (PerkinElmer). β-Galactosidase activity was measured using chlorophenol red-β-D-galactopyranoside (Roche Applied Science) in a reaction buffer (60 mM Na_2_HPO_4_, 40 mM NaH_2_PO_4_, 1 mM MgSO_4_, 10 mM KCl, 50 mM 2-mercaptoethanol, pH 7.0) to normalize luciferase activity.

### Statistical analysis

All experiments were performed in triplicates, and statistical significance was determined using Student’s *t* test (paired, two-tailed) or two-way ANOVA followed by Bonferroni’s post hoc test. All data are expressed as means ± SD. Adjusted *p* values were calculated using Benjamini–Hochberg procedure.

## Data availability

All data are contained within the manuscript.

## Supporting information

This article contains supporting information.

## Conflict of interest

The authors declare that they have no conflicts of interest with the contents of this article.
