# Supplementary material for: MicroRNA-593-5p contributes to cell death following exposure to 1-methyl-4-phenylpyridinium by targeting PTEN-induced putative kinase 1
Source: J Biol Chem. 2023 Apr 14;299(5):104709. doi: 10.1016/j.jbc.2023.104709 (PMC10196868; doi:10.1016/j.jbc.2023.104709)
Supplement: Supporting Table S1 [file mmc1.pdf]

Table S1. List of total miRs with expression changes (log2FC) and statistical significance (log10 adjP).

Up-regulated miR	log2FC	log10(P)$^{-1}$	Down-regulated miR	log2FC	log10(P)$^{-1}$
hsa-miR-593*	3.81	1.943591	hsa-miR-188-5p	-0.00667	0.005815
hsa-miR-551b*	2	0.25036	hsa-miR-589*	-0.01333	0.029426
hsa-let-7g*	1.79	0.78907	hsa-miR-26b	-0.01333	0.013643
hsa-miR-573	1.72	0.804978	hsa-miR-2052	-0.01667	0.0068
hsa-miR-449b*	1.65	1.259283	hsa-miR-874	-0.01667	0.035854
hsa-miR-219-2-3p	1.65	0.836608	hsa-miR-30c	-0.02	0.012684
hsa-miR-3182	1.603333	0.844614	hsa-miR-4252	-0.02333	0.009191
hsa-miR-219-1-3p	1.603333	1.496222	hsa-miR-126*	-0.02333	0.021841
hsa-miR-1976	1.456667	0.248143	hsa-miR-1250	-0.02667	0.020633
hsa-miR-1231	1.433333	1.011854	hsa-miR-378*	-0.02667	0.008831
hsa-miR-4318	1.356667	0.503915	hsa-miR-99b*	-0.03333	0.017766
hsa-miR-323-5p	1.353333	0.350364	hsa-miR-22*	-0.03667	0.029108
hsa-let-7f-1*	1.336667	0.864694	hsa-miR-876-5p	-0.04333	0.071596
hsa-miR-487a	1.326667	0.449828	hsa-miR-320e	-0.04667	0.044382
hsa-miR-564	1.313333	0.559871	hsa-miR-1258	-0.05	0.027728
hsa-miR-135a	1.293333	1.591535	hsa-miR-181a-2*	-0.06	0.09947
hsa-miR-3200-5p	1.29	0.271896	hsa-miR-4291	-0.06667	0.036922
hsa-miR-4301	1.26	0.274909	hsa-miR-218-2*	-0.07	0.052031
hsa-miR-542-5p	1.26	0.24633	hsa-miR-199b-5p	-0.07	0.060998
hsa-miR-129-5p	1.22	0.333031	hsa-miR-24-2*	-0.07	0.091066
hsa-miR-219-5p	1.216667	0.68972	hsa-miR-550a*	-0.07667	0.029374
hsa-miR-1203	1.213333	0.351208	hsa-miR-29b-1*	-0.08	0.167502
hsa-miR-20a	1.203333	1.561014	hsa-miR-411	-0.08	0.055099
hsa-miR-106a*	1.176667	0.298282	hsa-miR-1269	-0.08333	0.041333
hsa-miR-342-3p	1.173333	0.277603	hsa-miR-152	-0.09	0.041452
hsa-miR-548s	1.163333	0.353492	hsa-miR-129-3p	-0.09	0.079128
hsa-miR-138	1.16	0.353517	hsa-miR-500a	-0.09333	0.075022
hsa-miR-4326	1.16	0.306714	hsa-miR-3613-3p	-0.09667	0.180745
hsa-miR-3130-5p	1.153333	0.349571	hsa-miR-502-5p	-0.1	0.067797
hsa-miR-3907	1.09	0.356938	hsa-miR-1281	-0.10333	0.028925
hsa-miR-937	1.076667	0.502178	hsa-miR-655	-0.10667	0.049245
hsa-miR-23a*	1.06	0.248523	hsa-miR-137	-0.10667	0.079518
hsa-miR-1303	1.036667	0.367835	hsa-miR-33a*	-0.11	0.078103
hsa-miR-4302	1.033333	0.167414	hsa-miR-125b-2*	-0.11667	0.078671
hsa-miR-31	1.03	0.328727	hsa-miR-499	-0.12333	0.08443
hsa-miR-591	1.02	0.651791	hsa-miR-1908	-0.12667	0.192464
hsa-miR-548v	1.013333	0.49286	hsa-miR-9*	-0.13333	0.201458
hsa-miR-331-3p	1.01	0.220138	hsa-miR-193a-5p	-0.13667	0.091969
hsa-miR-1296	1.003333	0.317611	hsa-let-7e*	-0.14667	0.052848
hsa-miR-582-3p	1	0.22194	hsa-miR-153	-0.14667	0.122241
hsa-miR-298	0.993333	0.372718	hsa-miR-1266	-0.15	0.108748
hsa-miR-3918	0.99	0.275631	hsa-let-7b*	-0.15	0.065925
hsa-miR-590-5p	0.986667	0.862595	hsa-miR-3115	-0.15667	0.196966
hsa-miR-550a	0.983333	0.379538	hsa-miR-25*	-0.16	0.094854

hsa-miR-629	0.976667	0.434773	hsa-miR-1271	-0.16	0.070723
hsa-miR-27b	0.963333	0.501479	hsa-miR-105	-0.17667	0.09598
hsa-miR-1910	0.963333	0.199136	hsa-miR-374a*	-0.18667	0.088185
hsa-miR-1233	0.96	0.201984	hsa-miR-196a	-0.19333	0.093125
hsa-miR-191	0.96	0.354814	hsa-miR-370	-0.19333	0.070766
hsa-miR-320b	0.956667	0.84826	hsa-miR-7-2*	-0.2	0.090148
hsa-miR-1227	0.95	0.219391	hsa-miR-1280	-0.2	0.093323
hsa-miR-483-5p	0.95	0.353321	hsa-miR-154*	-0.20333	0.110027
hsa-miR-139-5p	0.946667	0.204008	hsa-miR-661	-0.20333	0.077155
hsa-miR-3687	0.94	0.332444	hsa-miR-1180	-0.20333	0.104789
hsa-miR-362-3p	0.94	0.36565	hsa-miR-548aa	-0.22333	0.065216
hsa-miR-1	0.93	0.381195	hsa-miR-592	-0.22667	0.142335
hsa-miR-154	0.923333	0.345061	hsa-miR-99a*	-0.23667	0.091183
hsa-miR-501-5p	0.913333	0.320448	hsa-miR-103a-2*	-0.24	0.065417
hsa-miR-16	0.906667	0.37512	hsa-miR-3065-5p	-0.24333	0.105764
hsa-let-7b	0.896667	0.249853	hsa-miR-424*	-0.25667	0.140479
hsa-miR-195*	0.893333	0.380449	hsa-miR-629*	-0.26667	0.09863
hsa-miR-374a	0.89	0.572323	hsa-miR-1248	-0.26667	0.119939
hsa-miR-1914	0.88	0.352042	hsa-miR-627	-0.29	0.200462
hsa-miR-218	0.86	0.34736	hsa-miR-422a	-0.29333	0.355699
hsa-miR-3065-3p	0.856667	0.224452	hsa-miR-454*	-0.31	0.102223
hsa-miR-33a	0.846667	0.570259	hsa-miR-7-1*	-0.31	0.099164
hsa-miR-566	0.843333	0.150966	hsa-miR-378b	-0.31667	0.108025
hsa-miR-451	0.84	0.272413	hsa-miR-4258	-0.32	0.052506
hsa-miR-3926	0.833333	0.325379	hsa-miR-1270	-0.32667	0.107535
hsa-miR-3200-3p	0.83	0.229107	hsa-miR-708*	-0.35	0.202301
hsa-miR-382	0.826667	0.713088	hsa-miR-3154	-0.36333	0.203566
hsa-miR-501-3p	0.826667	0.401937	hsa-miR-608	-0.37333	0.465546
hsa-miR-98	0.823333	0.32322	hsa-miR-3647-3p	-0.39333	0.098638
hsa-miR-380*	0.806667	0.203014	hsa-miR-625*	-0.40667	0.149848
hsa-miR-490-5p	0.8	0.219837	hsa-miR-142-3p	-0.41	0.173935
hsa-miR-339-3p	0.796667	0.506876	hsa-miR-29a*	-0.42333	0.246505
hsa-miR-1183	0.793333	0.351258	hsa-miR-16-1*	-0.43667	0.274114
hsa-miR-99a	0.79	0.387247	hsa-miR-32*	-0.45	0.194589
hsa-miR-3607-3p	0.783333	0.353941	hsa-miR-26b*	-0.45333	0.20994
hsa-miR-493	0.78	0.71003	hsa-miR-448	-0.45333	0.54205
hsa-miR-212	0.776667	0.3606	hsa-miR-92a-1*	-0.45667	0.218874
hsa-miR-301a	0.773333	0.507578	hsa-miR-3679-5p	-0.46333	0.145472
hsa-miR-653	0.773333	0.296084	hsa-miR-320d	-0.47333	0.251273
hsa-miR-1298	0.77	0.630337	hsa-miR-3654	-0.48	0.19947
hsa-miR-29b	0.77	0.19574	hsa-miR-664*	-0.48667	0.175894
hsa-miR-637	0.76	0.235398	hsa-miR-331-5p	-0.51667	0.215225
hsa-miR-148b	0.76	0.76924	hsa-miR-1285	-0.52333	0.25212
hsa-miR-505	0.76	0.17613	hsa-miR-4296	-0.55	0.101961
hsa-miR-191*	0.756667	0.76891	hsa-miR-93*	-0.55667	0.218342
hsa-miR-548d-3p	0.756667	0.244542	hsa-miR-720	-0.57667	0.213695
hsa-miR-941	0.753333	0.364108	hsa-miR-15b*	-0.58667	0.210571

hsa-let-7d	0.743333	0.398307
hsa-miR-196b*	0.74	0.201919
hsa-miR-29a	0.723333	0.22237
hsa-miR-486-5p	0.713333	0.717998
hsa-let-7g	0.713333	0.228913
hsa-miR-21*	0.703333	0.376249
hsa-miR-27a*	0.703333	0.165292
hsa-miR-942	0.703333	0.469058
hsa-miR-103a	0.696667	0.252725
hsa-miR-25	0.696667	0.212798
hsa-miR-30b*	0.693333	0.248423
hsa-miR-1537	0.693333	0.162659
hsa-miR-3117-3p	0.693333	0.467638
hsa-miR-24	0.693333	0.674033
hsa-miR-203	0.683333	0.132206
hsa-let-7d*	0.683333	0.358198
hsa-miR-34c-3p	0.68	0.329364
hsa-miR-92b	0.67	0.370064
hsa-miR-146b-5p	0.67	0.243041
hsa-miR-138-1*	0.663333	0.245992
hsa-miR-1471	0.66	0.250917
hsa-miR-2113	0.656667	0.49309
hsa-miR-876-3p	0.656667	0.247166
hsa-miR-619	0.656667	0.674221
hsa-miR-376b	0.653333	0.197064
hsa-miR-548w	0.65	0.252381
hsa-miR-20b*	0.646667	0.570283
hsa-miR-100	0.646667	0.210947
hsa-miR-2355-3p	0.643333	0.218541
hsa-miR-374b*	0.636667	0.108708
hsa-miR-675*	0.636667	0.596412
hsa-miR-1912	0.633333	0.717931
hsa-miR-770-5p	0.633333	0.378485
hsa-miR-4279	0.62	0.512009
hsa-let-7f	0.62	0.122063
hsa-miR-187	0.613333	0.50009
hsa-miR-1307	0.613333	0.175039
hsa-miR-1184	0.61	0.329345
hsa-miR-378	0.606667	0.473102
hsa-miR-489	0.603333	0.329631
hsa-miR-3186-3p	0.6	0.277742
hsa-miR-361-3p	0.596667	0.620525
hsa-miR-548k	0.593333	0.227247
hsa-miR-95	0.593333	0.439024
hsa-miR-143	0.59	0.533529
hsa-miR-30c-1*	0.59	0.251227
hsa-miR-1247	0.586667	0.171229

hsa-miR-101*	-0.61333	0.169785
hsa-miR-670	-0.62	0.203071
hsa-miR-335*	-0.65333	0.347878
hsa-miR-299-5p	-0.65667	0.348346
hsa-miR-2110	-0.67333	0.294443
hsa-miR-138-2*	-0.68	0.105861
hsa-miR-497*	-0.68	0.168641
hsa-miR-221	-0.7	0.248759
hsa-miR-7	-0.70333	0.104766
hsa-miR-514b-3p	-0.70333	0.679396
hsa-miR-539	-0.72667	0.370998
hsa-miR-1260	-0.73	0.250457
hsa-miR-1539	-0.73667	0.245321
hsa-miR-26a-1*	-0.74	0.25158
hsa-miR-23b*	-0.80333	0.314111
hsa-miR-4274	-0.80667	0.30118
hsa-miR-1538	-0.81333	0.250106
hsa-miR-19b-1*	-0.82	0.21133
hsa-miR-631	-0.82333	0.275296
hsa-miR-377	-0.83333	0.315563
hsa-miR-4316	-0.84667	0.78629
hsa-miR-381	-0.86	0.349954
hsa-miR-19a*	-0.86	0.208842
hsa-miR-1287	-0.91	0.430355
hsa-miR-2278	-0.95	0.248051
hsa-miR-1286	-0.99	0.268686
hsa-miR-1825	-1.07333	0.302333
hsa-miR-18b	-1.09333	0.800778
hsa-miR-570	-1.25	0.330594
hsa-miR-4262	-1.28333	0.331356
hsa-miR-26a-2*	-1.46667	0.641717

hsa-miR-491-5p	0.586667	0.132365
hsa-miR-330-5p	0.586667	0.353899
hsa-miR-455-5p	0.583333	0.236136
hsa-miR-877	0.583333	0.347494
hsa-miR-4289	0.58	0.11685
hsa-miR-590-3p	0.573333	0.943877
hsa-miR-3909	0.57	0.173793
hsa-miR-145*	0.563333	0.27166
hsa-miR-29c	0.56	0.195913
hsa-miR-769-5p	0.556667	0.327651
hsa-miR-376c	0.546667	0.460781
hsa-miR-296-5p	0.543333	0.171245
hsa-let-7i	0.543333	0.179451
hsa-miR-107	0.54	0.099053
hsa-miR-34b	0.54	0.352072
hsa-miR-548e	0.536667	0.529577
hsa-miR-484	0.533333	0.247642
hsa-miR-190	0.526667	1.531328
hsa-miR-662	0.516667	0.19362
hsa-miR-132*	0.51	0.269236
hsa-miR-744*	0.51	0.161563
hsa-miR-1301	0.51	0.208863
hsa-miR-96	0.51	0.217584
hsa-miR-34a*	0.506667	0.136288
hsa-miR-194	0.503333	0.376262
hsa-miR-379	0.503333	0.269196
hsa-miR-195	0.503333	0.326832
hsa-miR-132	0.496667	0.271735
hsa-miR-28-5p	0.496667	0.475118
hsa-miR-3141	0.493333	0.21981
hsa-miR-149	0.49	0.316804
hsa-miR-646	0.486667	0.097798
hsa-miR-125a-5p	0.483333	0.174329
hsa-miR-551a	0.48	0.11998
hsa-miR-130a	0.47	0.271134
hsa-miR-425	0.463333	0.245798
hsa-miR-134	0.463333	0.251582
hsa-miR-615-3p	0.46	0.277738
hsa-miR-128	0.46	0.242218
hsa-miR-30d	0.46	0.404182
hsa-miR-423-3p	0.456667	0.249514
hsa-miR-4253	0.453333	0.517907
hsa-miR-19b	0.453333	0.20285
hsa-miR-200c	0.453333	0.352354
hsa-miR-3682-3p	0.45	0.201142
hsa-miR-628-3p	0.45	0.105826
hsa-miR-502-3p	0.446667	0.501373

hsa-miR-500a*	0.446667	0.163421
hsa-miR-488	0.443333	0.079002
hsa-miR-610	0.44	0.782964
hsa-miR-596	0.44	0.254086
hsa-miR-4300	0.436667	0.244863
hsa-miR-376a	0.43	0.370141
hsa-miR-660	0.426667	0.341296
hsa-miR-885-5p	0.426667	0.361841
hsa-miR-29b-2*	0.426667	0.140615
hsa-miR-186	0.426667	0.280043
hsa-miR-454	0.423333	0.091866
hsa-miR-214	0.423333	0.175334
hsa-miR-101	0.42	0.223542
hsa-miR-18a*	0.42	0.202299
hsa-miR-181c	0.416667	0.102458
hsa-miR-3174	0.416667	0.210995
hsa-miR-15b	0.413333	0.19481
hsa-miR-4265	0.41	0.431241
hsa-miR-34c-5p	0.406667	0.205021
hsa-miR-375	0.403333	0.357092
hsa-miR-140-5p	0.4	0.2005
hsa-miR-3651	0.4	0.176928
hsa-miR-920	0.4	0.132905
hsa-miR-140-3p	0.396667	0.351894
hsa-miR-1306	0.393333	0.253673
hsa-miR-222	0.393333	0.17982
hsa-miR-146a	0.39	0.220747
hsa-miR-22	0.39	0.177112
hsa-miR-1253	0.386667	0.145618
hsa-miR-106b*	0.386667	0.293726
hsa-miR-362-5p	0.386667	0.350591
hsa-miR-106b	0.386667	0.09006
hsa-miR-199b-3p	0.383333	0.099415
hsa-miR-485-3p	0.383333	0.069987
hsa-miR-328	0.383333	0.098347
hsa-miR-3131	0.383333	0.144645
hsa-miR-3655	0.38	0.203025
hsa-miR-34a	0.373333	0.177714
hsa-miR-877*	0.37	0.165742
hsa-miR-575	0.37	0.348575
hsa-miR-183*	0.366667	0.238617
hsa-miR-4323	0.366667	0.275115
hsa-miR-23b	0.366667	0.273931
hsa-miR-30b	0.363333	0.17972
hsa-miR-563	0.36	0.250372
hsa-miR-532-5p	0.36	0.197937
hsa-miR-4286	0.356667	0.136658

hsa-miR-329	0.353333	0.107876
hsa-miR-361-5p	0.353333	0.252286
hsa-miR-1238	0.353333	0.204051
hsa-miR-1304	0.35	0.092362
hsa-miR-548d-5p	0.35	0.238267
hsa-miR-9	0.35	0.168362
hsa-miR-186*	0.346667	0.087796
hsa-let-7f-2*	0.346667	0.969547
hsa-miR-32	0.346667	0.212257
hsa-miR-141	0.346667	0.107938
hsa-miR-548c-5p	0.343333	0.140609
hsa-miR-551b	0.343333	0.244643
hsa-miR-340	0.336667	0.203796
hsa-miR-3714	0.333333	0.093376
hsa-miR-1224-5p	0.333333	0.118867
hsa-miR-624*	0.33	0.167201
hsa-miR-23a	0.33	0.153338
hsa-miR-93	0.326667	0.105745
hsa-miR-210	0.326667	0.097814
hsa-miR-214*	0.323333	0.195711
hsa-miR-373*	0.32	0.16653
hsa-miR-4299	0.32	0.203363
hsa-miR-935	0.32	0.208806
hsa-miR-92b*	0.316667	0.178465
hsa-miR-151-5p	0.313333	0.18084
hsa-miR-338-3p	0.313333	0.102017
hsa-miR-4263	0.313333	0.213798
hsa-miR-30e	0.306667	0.177325
hsa-miR-15a	0.306667	0.11908
hsa-miR-345	0.303333	0.106056
hsa-miR-664	0.303333	0.175813
hsa-miR-92a	0.303333	0.350087
hsa-miR-604	0.296667	0.221106
hsa-miR-30a*	0.296667	0.09798
hsa-miR-450a	0.293333	0.083201
hsa-miR-139-3p	0.293333	0.650919
hsa-miR-3939	0.293333	0.220466
hsa-miR-532-3p	0.29	0.092769
hsa-miR-409-3p	0.29	0.145705
hsa-miR-324-5p	0.29	0.130453
hsa-miR-542-3p	0.29	0.069284
hsa-miR-449a	0.29	0.067672
hsa-miR-495	0.286667	0.181565
hsa-miR-200b	0.286667	0.06979
hsa-miR-3180-3p	0.286667	0.274227
hsa-miR-548i	0.283333	0.129435
hsa-miR-196b	0.28	0.157467

hsa-let-7a	0.28	0.079475
hsa-miR-23c	0.276667	0.254445
hsa-miR-24-1*	0.276667	0.199529
hsa-miR-339-5p	0.27	0.14821
hsa-let-7i*	0.266667	0.143605
hsa-miR-873	0.266667	0.111596
hsa-miR-27b*	0.266667	0.084916
hsa-miR-921	0.266667	0.122498
hsa-miR-598	0.263333	0.069997
hsa-miR-543	0.263333	0.119598
hsa-miR-1277	0.263333	0.246899
hsa-miR-330-3p	0.26	0.109681
hsa-miR-3913-5p	0.253333	0.070141
hsa-miR-505*	0.253333	0.053532
hsa-miR-1249	0.253333	0.678999
hsa-miR-185	0.25	0.099472
hsa-miR-346	0.246667	0.087106
hsa-miR-125a-3p	0.246667	0.21
hsa-miR-3676	0.243333	0.080248
hsa-miR-1291	0.243333	0.176852
hsa-miR-181c*	0.243333	0.07974
hsa-miR-431	0.236667	0.145809
hsa-miR-183	0.236667	0.065773
hsa-let-7a*	0.236667	0.176195
hsa-miR-548o	0.236667	0.051616
hsa-miR-708	0.233333	0.06499
hsa-miR-3622a-5p	0.23	0.106333
hsa-miR-943	0.23	0.050965
hsa-miR-340*	0.23	0.069424
hsa-miR-30a	0.23	0.177733
hsa-miR-574-3p	0.226667	0.200845
hsa-miR-17*	0.226667	0.143382
hsa-miR-548b-5p	0.223333	0.172272
hsa-miR-27a	0.22	0.093835
hsa-miR-1267	0.22	0.141385
hsa-miR-15a*	0.22	0.052751
hsa-miR-16-2*	0.216667	0.060175
hsa-miR-2355-5p	0.216667	0.090034
hsa-miR-182	0.216667	0.040754
hsa-miR-767-3p	0.213333	0.091754
hsa-miR-17	0.21	0.051886
hsa-miR-425*	0.206667	0.091277
hsa-miR-374c	0.206667	0.100021
hsa-miR-99b	0.206667	0.351167
hsa-miR-129*	0.203333	0.103814
hsa-miR-215	0.2	0.178995
hsa-miR-130b	0.2	0.09131

hsa-miR-410	0.196667	0.066839
hsa-miR-148a	0.196667	0.062832
hsa-miR-197	0.193333	0.059422
hsa-miR-193a-3p	0.193333	0.051999
hsa-miR-320a	0.19	0.574735
hsa-miR-887	0.19	0.350755
hsa-miR-3653	0.19	0.092219
hsa-miR-548b-3p	0.186667	0.091745
hsa-miR-651	0.186667	0.178334
hsa-miR-19a	0.183333	0.102452
hsa-miR-145	0.18	0.064737
hsa-miR-10a	0.18	0.070213
hsa-miR-490-3p	0.176667	0.143666
hsa-miR-369-3p	0.176667	0.041348
hsa-miR-181b	0.173333	0.099634
hsa-miR-1237	0.17	0.119784
hsa-miR-424	0.17	0.053529
hsa-miR-516b	0.17	0.091194
hsa-miR-3125	0.17	0.052919
hsa-miR-324-3p	0.163333	0.053594
hsa-miR-199a-5p	0.163333	0.047821
hsa-miR-125b	0.163333	0.054367
hsa-miR-3908	0.163333	0.098057
hsa-miR-28-3p	0.163333	0.069333
hsa-miR-483-3p	0.16	0.070585
hsa-miR-18a	0.16	0.05155
hsa-let-7c	0.16	0.047523
hsa-miR-1276	0.16	0.069865
hsa-miR-363	0.156667	0.051767
hsa-miR-296-3p	0.153333	0.20116
hsa-miR-193b	0.146667	0.070048
hsa-miR-1179	0.146667	0.066111
hsa-miR-33b	0.146667	0.070082
hsa-miR-659	0.146667	0.050309
hsa-miR-577	0.146667	0.036112
hsa-miR-1226	0.143333	0.176403
hsa-miR-135b	0.143333	0.110019
hsa-miR-487b	0.14	0.181828
hsa-miR-20a*	0.136667	0.270183
hsa-miR-151-3p	0.136667	0.066148
hsa-miR-589	0.136667	0.071213
hsa-miR-579	0.136667	0.040591
hsa-miR-891a	0.133333	0.119072
hsa-miR-767-5p	0.133333	0.052683
hsa-miR-3176	0.13	0.081875
hsa-miR-617	0.13	0.051448
hsa-miR-26a	0.13	0.051514

hsa-miR-423-5p	0.126667	0.187179
hsa-miR-576-3p	0.123333	0.091472
hsa-miR-1260b	0.12	0.048793
hsa-miR-148b*	0.12	0.024775
hsa-miR-1228*	0.113333	0.075469
hsa-miR-21	0.113333	0.042161
hsa-miR-744	0.113333	0.097104
hsa-miR-766	0.113333	0.053347
hsa-miR-181a	0.11	0.070757
hsa-miR-365	0.11	0.032312
hsa-miR-326	0.11	0.053101
hsa-miR-497	0.106667	0.146482
hsa-miR-10b*	0.103333	0.058767
hsa-miR-20b	0.103333	0.043955
hsa-miR-3161	0.103333	0.063755
hsa-miR-545*	0.103333	0.052516
hsa-miR-380	0.103333	0.099048
hsa-miR-29c*	0.1	0.090861
hsa-miR-582-5p	0.1	0.051484
hsa-miR-576-5p	0.1	0.049779
hsa-miR-3652	0.096667	0.070282
hsa-miR-1909*	0.096667	0.066541
hsa-miR-130b*	0.096667	0.087836
hsa-miR-652	0.096667	0.066956
hsa-miR-1290	0.09	0.017818
hsa-miR-30d*	0.086667	0.109069
hsa-miR-455-3p	0.086667	0.162504
hsa-miR-192	0.08	0.047248
hsa-miR-1911*	0.08	0.093245
hsa-miR-181d	0.08	0.061179
hsa-miR-585	0.076667	0.031536
hsa-miR-3607-5p	0.073333	0.060818
hsa-miR-1252	0.073333	0.05317
hsa-miR-376a*	0.066667	0.033906
hsa-miR-3910	0.066667	0.029405
hsa-miR-628-5p	0.063333	0.059734
hsa-miR-126	0.063333	0.031252
hsa-miR-301b	0.063333	0.029406
hsa-miR-3618	0.06	0.025054
hsa-miR-124*	0.056667	0.031504
hsa-miR-676*	0.056667	0.017514
hsa-miR-30e*	0.053333	0.051271
hsa-miR-342-5p	0.053333	0.024314
hsa-miR-3647-5p	0.043333	0.040598
hsa-miR-1207-5p	0.04	0.012515
hsa-miR-545	0.04	0.052138
hsa-miR-193b*	0.036667	0.016024

hsa-miR-1261	0.03	0.011903
hsa-miR-10b	0.03	0.110151
hsa-miR-3185	0.03	0.017173
hsa-miR-3916	0.023333	0.018493
hsa-let-7e	0.02	0.00956
hsa-miR-124	0.013333	0.005891
hsa-miR-421	0.01	0.005791
hsa-miR-181a*	0.006667	0.005413
hsa-miR-188-3p	0.003333	0.001947
