# Supplementary figures and images for: MicroRNA-593-5p contributes to cell death following exposure to 1-methyl-4-phenylpyridinium by targeting PTEN-induced putative kinase 1
Source: J Biol Chem. 2023 Apr 14;299(5):104709. doi: 10.1016/j.jbc.2023.104709 (PMC10196868; doi:10.1016/j.jbc.2023.104709)

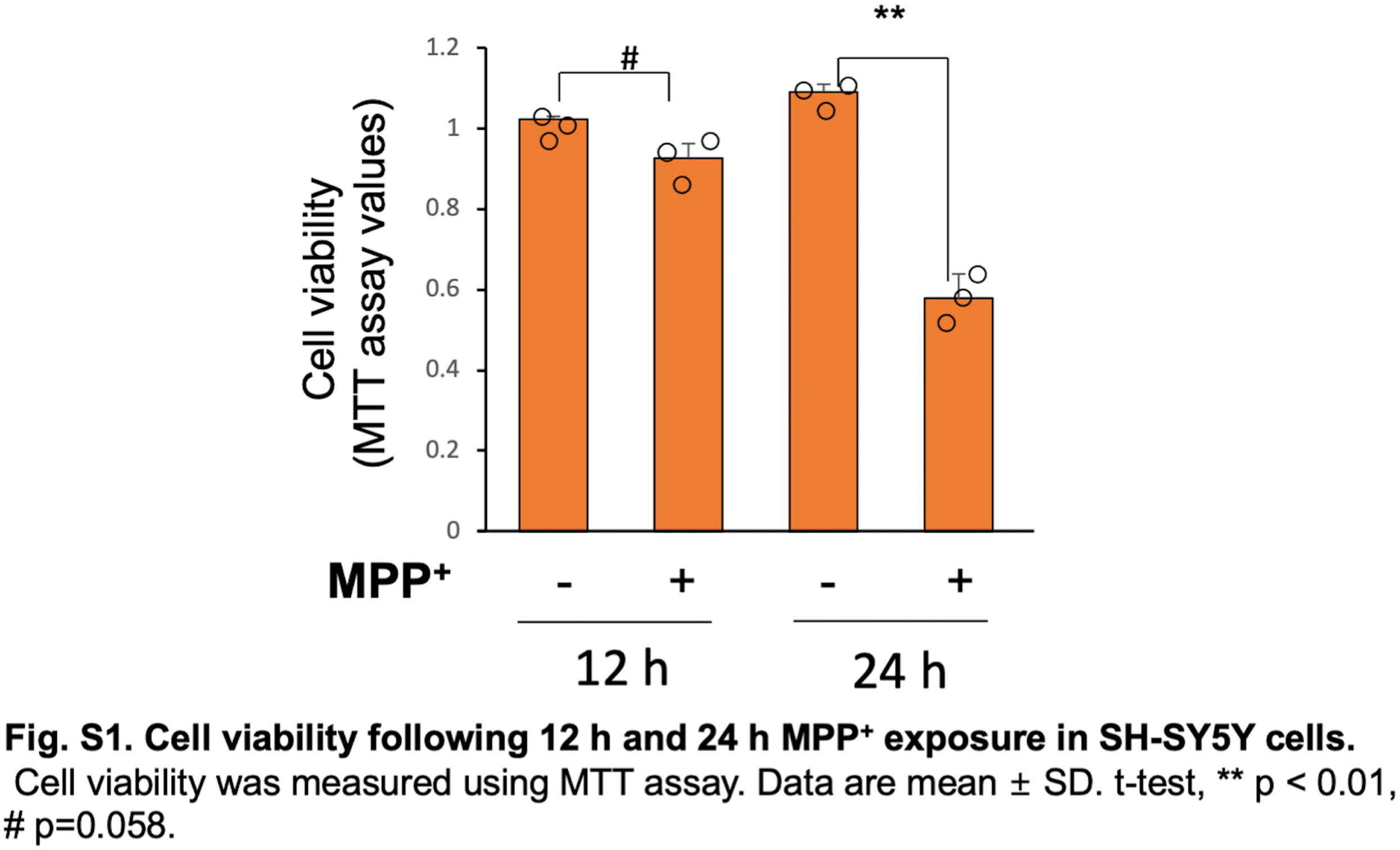

Supplement: Supporting Figure S1 [file figs1.jpg]

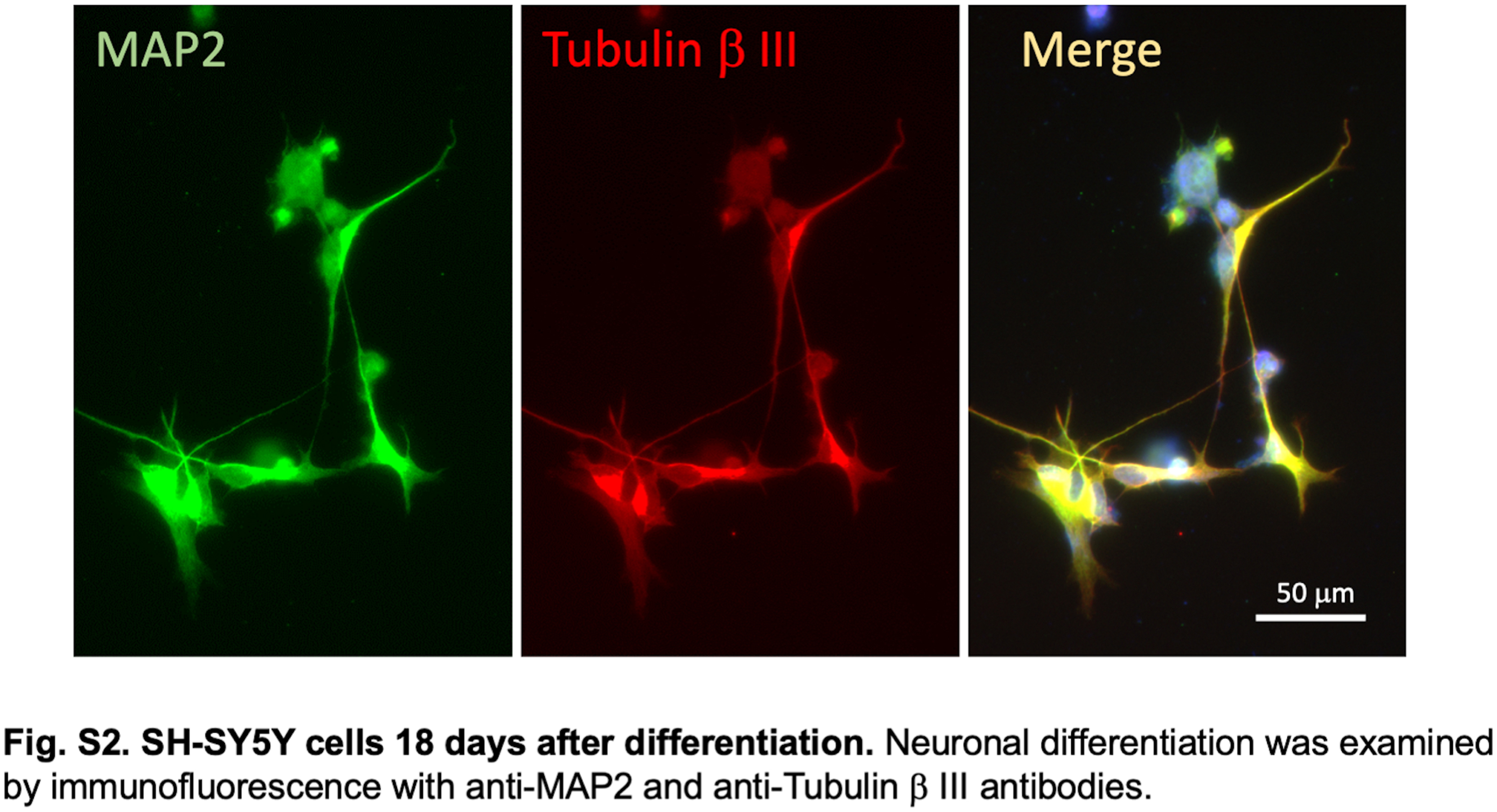

Supplement: Supporting Figure S2 [file figs2.jpg]

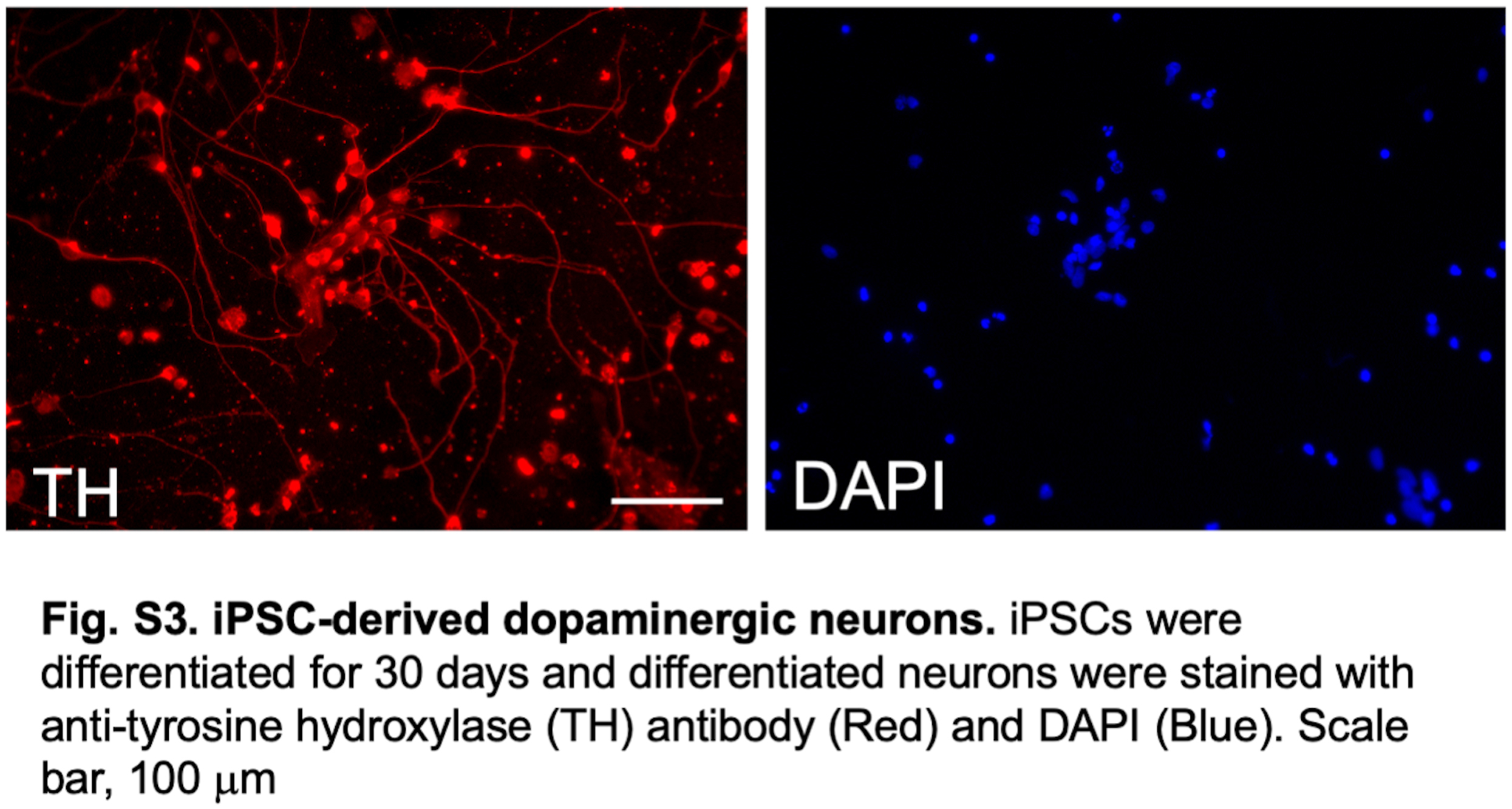

Supplement: Supporting Figure S3 [file figs3.jpg]

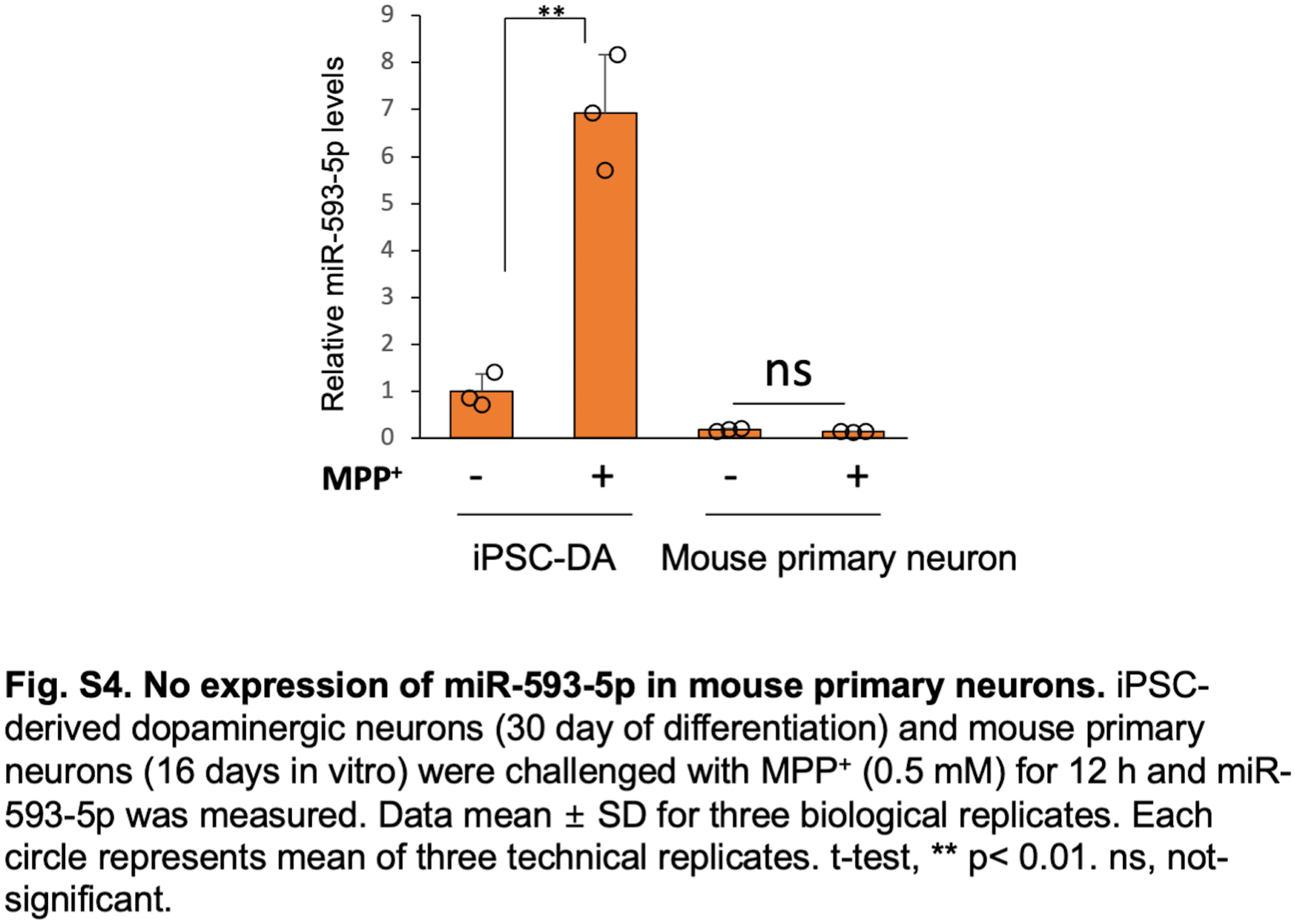

Supplement: Supporting Figure S4 [file figs4.jpg]

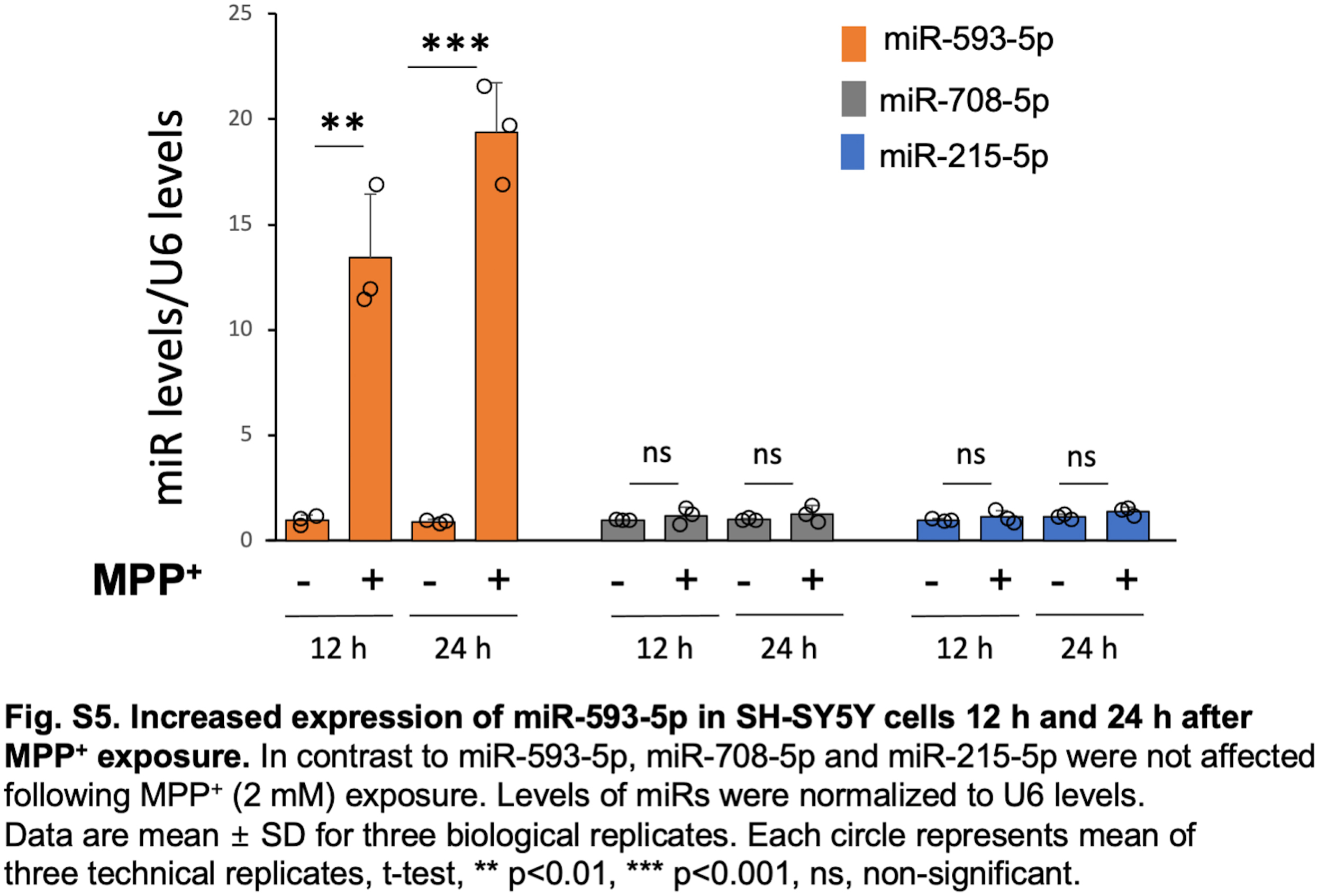

Supplement: Supporting Figure S5 [file figs5.jpg]

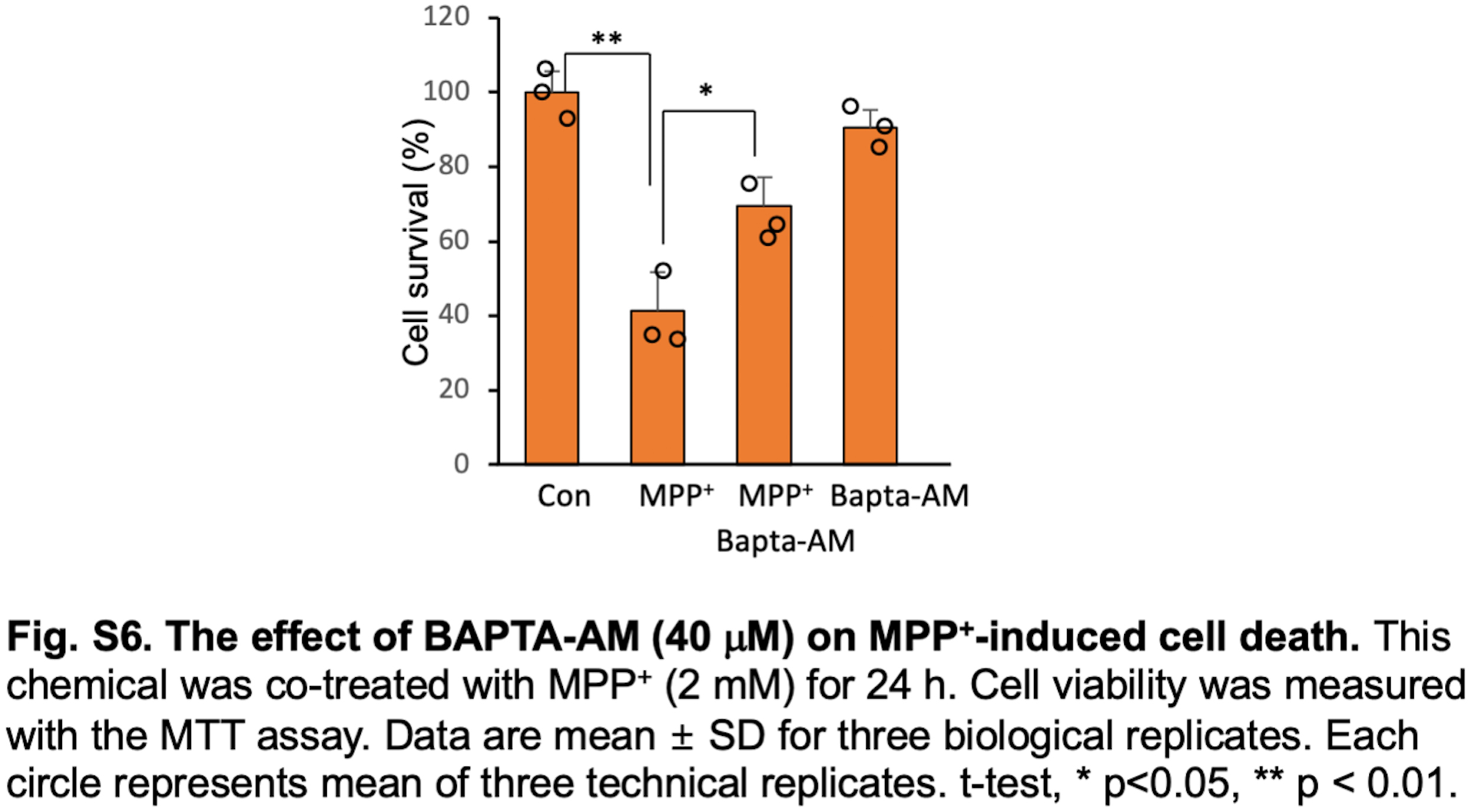

Supplement: Supporting Figure S6 [file figs6.jpg]

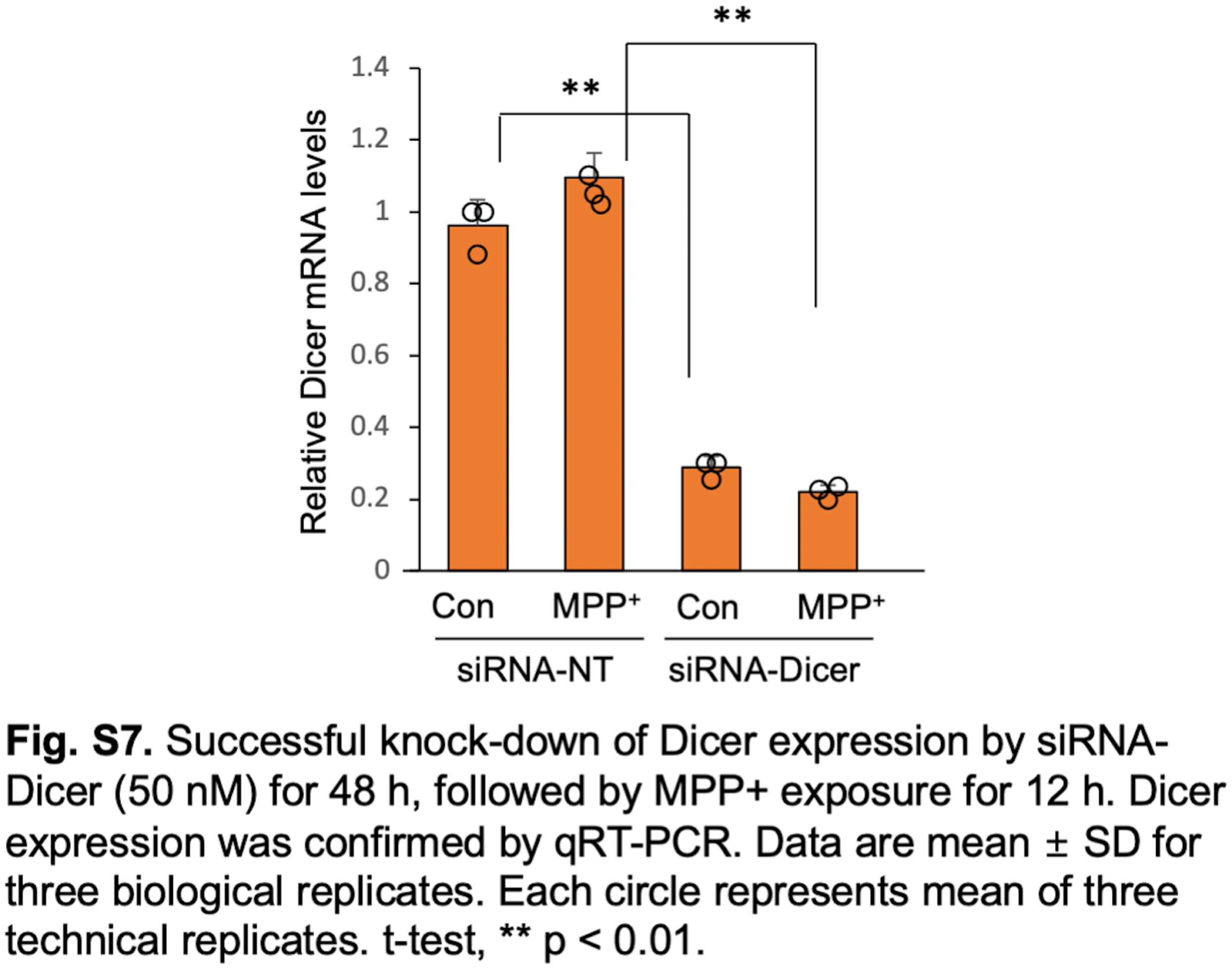

Supplement: Supporting Figure S7 [file figs7.jpg]

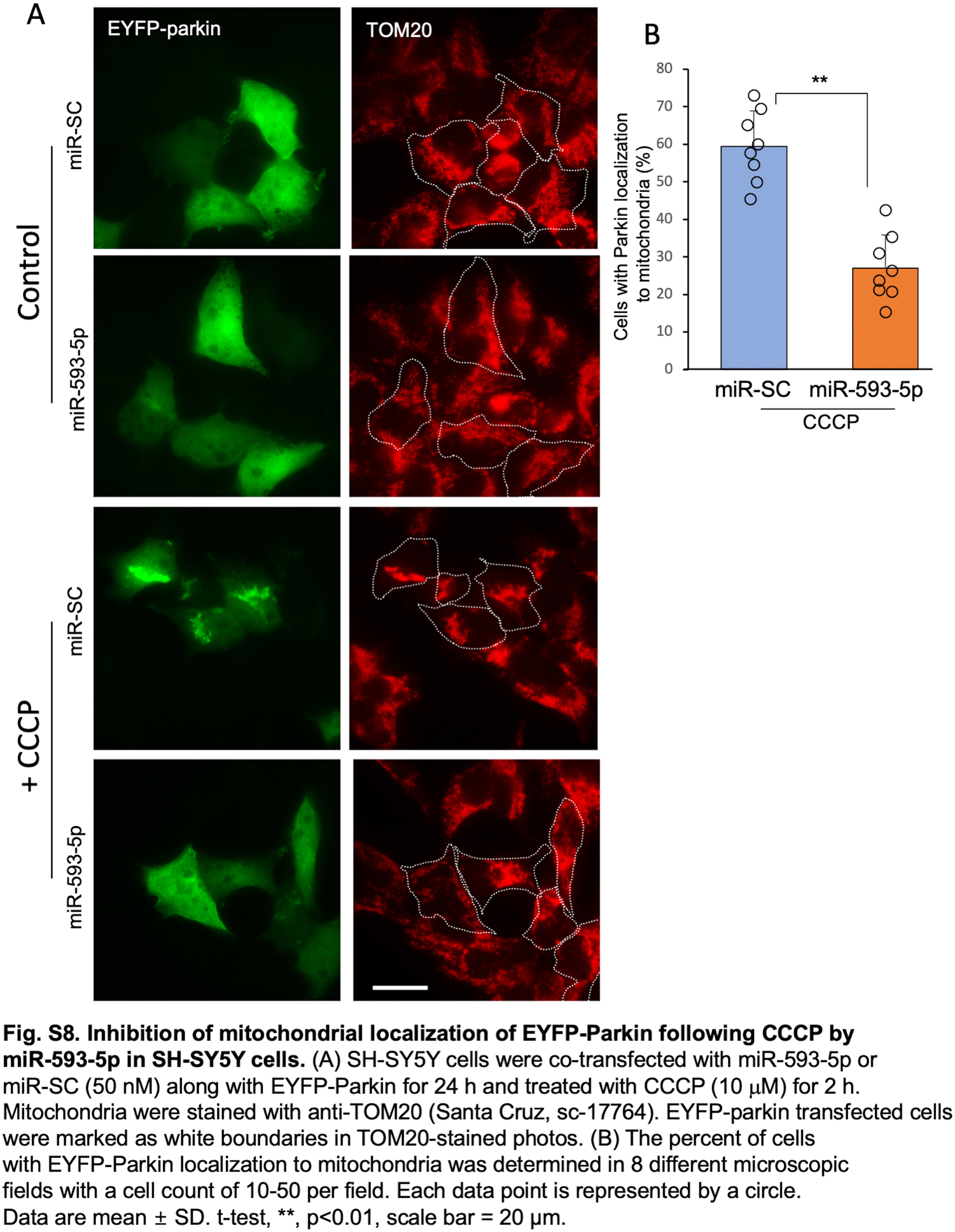

Supplement: Supporting Figure S8 [file figs8.jpg]

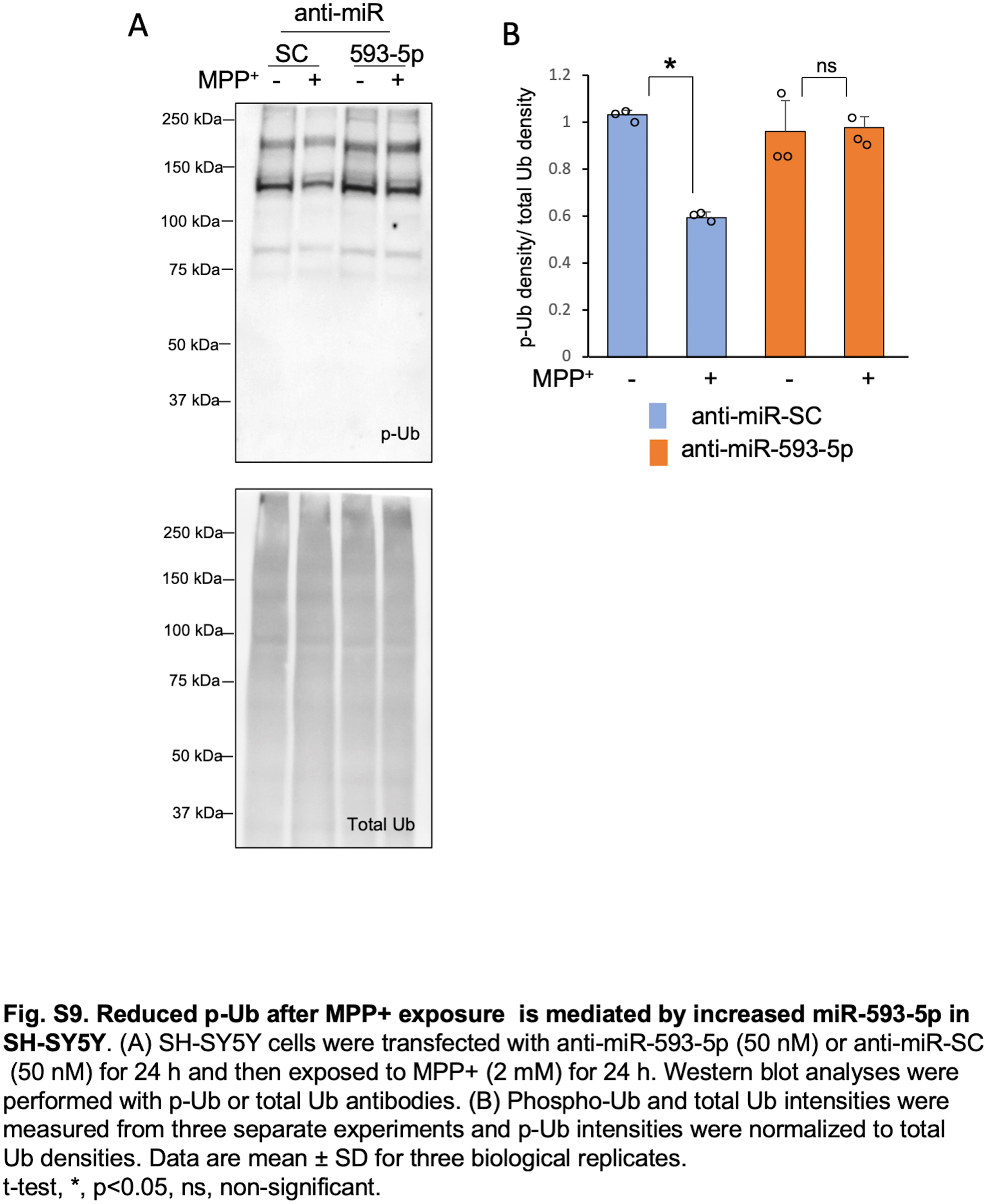

Supplement: Supporting Figure S9 [file figs9.jpg]
